# Identification of a JAK–STAT–miR155HG positive feedback loop in regulating natural killer (NK) cells proliferation and effector functions

**DOI:** 10.1016/j.apsb.2025.02.034

**Published:** 2025-03-02

**Authors:** Songyang Li, Yongjie Liu, Xiaofeng Yin, Yao Yang, Xinjia Liu, Jiaxing Qiu, Qinglan Yang, Yana Li, Zhiguo Tan, Hongyan Peng, Peiwen Xiong, Shuting Wu, Lanlan Huang, Xiangyu Wang, Sulai Liu, Yuxing Gong, Yuan Gao, Lingling Zhang, Junping Wang, Yafei Deng, Zhaoyang Zhong, Youcai Deng

**Affiliations:** a Pediatrics Research Institute of Hunan Province, the Affiliated Children's Hospital of Xiangya School of Medicine, Central South University (Hunan Children's Hospital), Changsha 410007, China; b Department of Clinical Hematology, College of Pharmacy and Laboratory Medicine Science, Army Medical University, Chongqing 400038, China; c Department of Pharmacy, the General Hospital of Western Theater Command of PLA, Chengdu 610083, China; d The School of Pediatrics, Hengyang Medical School, University of South China (Hunan Children's Hospital), Changsha 410007, China; e Department of Biological Sciences, Columbia University, NY 10027, USA; f Department of Hepatobiliary Surgery, Hunan Provincial People's Hospital (the First Affiliated Hospital of Hunan Normal University), Changsha 410005, China; g Translational Medicine Research Center, Shanxi Medical University, Taiyuan 030001, China; h Institute of Clinical Pharmacology, Anhui Medical University, Key Laboratory of Anti-inflammatory and Immune Medicine, Ministry of Education, Hefei 230032, China; i State Key Laboratory of Trauma and Chemical Poisoning, Institute of Combined Injury, Chongqing Engineering Research Center for Nanomedicine, College of Preventive Medicine, Army Medical University, Chongqing 400038, China; j The Fifth People's Hospital of Chongqing, Chongqing 400062, China

**Keywords:** NK cells, iPSC-NK cells, lncRNA, miR155HG, Competing endogenous RNA, miR-6756, JAK–STAT signaling pathway

## Abstract

The Janus kinase/signal transducers and activators of transcription (JAK–STAT) control natural killer (NK) cells development and cytotoxic functions, however, whether long non-coding RNAs (lncRNAs) are involved in this pathway remains unknown. We found that miR155HG was elevated in activated NK cells and promoted their proliferation and effector functions in both NK92 and induced-pluripotent stem cells (iPSCs)-derived NK (iPSC-NK) cells, without reliance on its derived miR-155 and micropeptide P155. Mechanistically, miR155HG bound to miR-6756 and relieved its repression of JAK3 expression, thereby promoting the JAK–STAT pathway and enhancing NK cell proliferation and function. Further investigations disclosed that upon cytokine stimulation, STAT3 directly interacts with miR155HG promoter and induces miR155HG transcription. Collectively, we identify a miR155HG-mediated positive feedback loop of the JAK–STAT signaling. Our study will also provide a power target regarding miR155HG for improving NK cell generation and effector function in the field of NK cell adoptive transfer therapy against cancer, especially iPSC-derived NK cells.

## Graphical abstract

miR155HG drives a JAK–STAT positive feedback loop in NK cells, and targeting it could boost NK cell generation and therapeutic efficacy in cancer immunotherapy.

**Figure:**
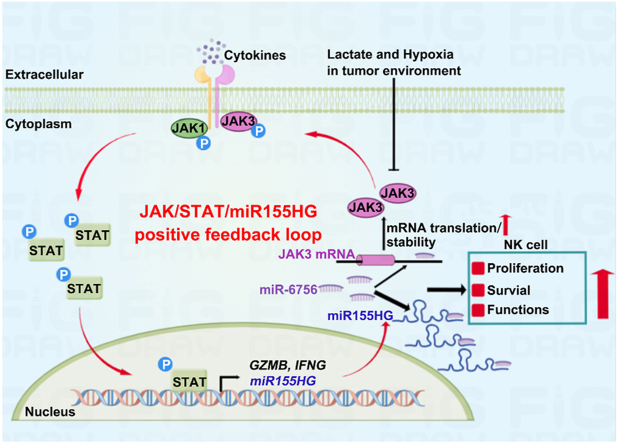

## 1 Introduction

Natural killer (NK) cells are the frontline guardians in the immune response to cancer and invading pathogens, providing strong antitumor effects and exhibiting favorable safety profiles in the field of allogeneic cancer immunotherapy^1^, ^2^, ^3^. Once triggered, NK cells not only unleash their cytotoxic contents, such as perforin and granzyme B, to induce direct lysis of cancer cells but also rapidly generate chemokines and cytokines like interferon-gamma (IFN-*γ*), thereby regulating the adaptive immune response. Additionally, enhanced NK effector activity is also triggered by CD16-mediated antibody-dependent cellular cytotoxicity signaling^2^^,^^4^. However, the clinical efficacy of NK cell therapy is constrained by their shorter lifespan and diminished activity within the immunosuppressive and cytokine-deficient tumor microenvironment (TME)^1^. Therefore, identifying the fundamental mechanisms that support NK cell homeostasis could deepen our comprehension of NK cell regulatory mechanisms and potentially uncover new therapeutic targets for enhancing cancer immunotherapy outcomes.

To date, the growth, longevity, and effectiveness of NK cells are thought to be significantly influenced by external cytokines and internal transcription factors^5^. The synergistic effects of various cytokines, including interleukin (IL)-2, IL-12, IL-15, IL-21, and interferons, are essential for NK cell development, maturation and homeostasis. The Janus kinase/signal transducers and activators of transcription (JAK–STAT) pathway is a prototypical example that regulates distinct aspects of NK cell biology, including cell identity, survival, cytotoxicity, type I Interferon response downstream of several cytokines^6^^,^^7^. It has been shown that defective JAK–STAT signaling in NK cells contributes to NK cell dysfunction and tumorigenesis, such as loss of JAK3 and STAT5b^8^^,^^9^. Although previous findings have reported that cytokine-inducible SH2-containing protein (CIS) acts in the negative feedback loops IL-15–JAK–STATs–CIS–IL-15^10,11^, the feedback loops of JAK–STATs are still largely unknown. Given the important role of the JAK–STAT pathway in NK cell survival and function, discovering new regulators that preserve its activity could reveal additional targets for cancer immunotherapy.

Long non-coding RNAs (lncRNAs) constitute a vast and heterogeneous group of non-coding RNAs that exceed 500 nucleotides in length^12^. LncRNAs exert important roles in various cell activities, including proliferation, metabolism and motility through RNA–RNA, RNA–DNA and RNA–protein interactions^13^, ^14^, ^15^. Some abundant lncRNAs, exemplified by the lncRNA PNUTS, bind to miRNA and act as miRNA sponges, thereby inhibiting the target-repressing function of miRNAs^13^. Presently, it remains to be determined whether lncRNA plays a regulatory role in the cytokine/JAK/STAT signaling pathway within NK cells.

In this study, we reveal a novel positive feedback loop in the JAK–STAT pathway, that is, cytokine/JAK/STAT signaling induces miR155HG transcription, which acts as a sponge for miR-6756, preventing the miR-6756-mediated repression of JAK3 expression and thereby enhancing the JAK–STAT signaling and thus NK cell activation and function.

## 2 Materials and methods

### 2.1 Reagents

The following reagents were used: IL-2 (#589106; BioLegend, San Diego, CA, USA), IL-3 (#AF-200-03-1MG; ThermoFisher Scientific, Waltham, MA, 02454, USA), IL-7 (#AF-200-07-1000UG; ThermoFisher Scientific), IL-12 (#573002; BioLegend), IL-15 (#570306; BioLegend), stem cell factor (SCF, #AF-300-07-500UG; ThermoFisher Scientific), fms-like tyrosine kinase receptor-3 ligand (FLT3L, #AF-300-19-1MG; ThermoFisher Scientific), Roswell Park Memorial Institute 1640 (RPMI 1640)/Dulbecco's modified Eagle's medium (DMEM) (Gibco, Grand Island, NY, USA), STEMdiff™ APEL™2 Medium (#05275; STEMCELL Technologies, Vancouver, Canada), TrypLE™ Select Enzyme (#12563029; STEMCELL Technologies), GlutaMAX™ DMEM (#10569010; ThermoFisher Scientific), GlutaMAX™ F12 (#31765035; ThermoFisher Scientific), fetal bovine serum (FBS; Biological Industries, Kibbutz Beit Haemek, Israel), PGM1 medium (#CA1007500; Cellapy, Beijing, China), Animal-Free Recombinant Human VEGF 165 (AF-100-20-250UG; Peprotech), Animal-Free Recombinant Human BMP4 (AF-120-05ET-250UG; Peprotech), Animal-Free Recombinant Human FGF-basic (AF-100-18B-100UG; Peprotech), Human/Murine/Rat Activin A (AF-120-14E-100UG; Peprotech), Human TPO (AF-300-18-1MG; Peprotech), Animal-Free Recombinant Human IL-6 (AF-200-06-100UG; Peprotech), Recombinant Human EPO (100-64-100UG; Peprotech), Animal-Free Recombinant Human IGF-II (AF-100-12-100UG; Peprotech), *β*-mercaptoethanol (#21985023; ThermoFisher Scientific), l-glutamine (#C0212; Beyotime, Shanghai, China), sodium selenite (#S5261; Sigma–Aldrich, Taufkirchen, Germany), l-ascorbic acid (#A4403; Sigma–Aldrich), cholamine (#E9508; Sigma–Aldrich), MEM Non-Essential Amino Acids Solution (#11140-050; Gibco), Ficoll (#17144003; GE Healthcare Bio-Sciences, Pittsburgh, PA, USA), Penicillin–Streptomycin (#15140122; ThermoFisher Scientific), human NK cell isolation kit (#130-098-185; Miltenyi Biotec, San Diego, CA, USA), Percoll (#17089101; GE Healthcare Bio-Sciences), Enzyme-linked immunosorbent assay (ELISA) kits (#430104; Biolegend), Protein transport inhibitor Golgi Plug (#555028; BD Bioscience, San Diego, CA, USA) and Golgi Stop (#554715; BD Bioscience), the Foxp3/transcription factor staining buffer set kit (#00-5523-00; eBioscience, San Diego, CA, USA), PMA (#00-4970-93; eBioscience), collagenase type II (#17101015; Gibco), collagenase type IV (#17104019; Gibco) and DNase I (#10104159001; Roche Diagnostics GmbH, Manheim, Germany), the apoptosis kit (#559763; BD Bioscience), CellTrace Violet Cell Proliferation Kit (#C34557; ThermoFisher Scientific), L-(+)-lactic acid (#L6402; Merck KGaA, Darmstadt, Germany), P155 (GenScript, Nanjing, China)^16^, NE-PER Nuclear and Cytoplasmic Extraction Reagents (#78833; ThermoFisher Scientific), RNA antisense purification (RAP) Kit (#Bes5103; BersinBio, Guangzhou, China), Chromatin immunoprecipitation (ChIP) assay Kit (#P2078; Beyotime), Ruxolitinib (S1378; Selleckchem, Houston, TX, USA), Stattic (S7024; Selleckchem), Actinomycin D (ActD, S8964; Selleckchem).

The following antibodies were used in Western blot: rabbit monoclonal antibodies (mAbs) against human phospho-Tyr980/981 of JAK3 (5031t), human phospho-Y705 of STAT3 (9145T), STAT3 (4904S), phospho-Y693 of STAT4 (4134S), STAT4 (2653S), phospho-Y694 of STAT5 (9359S), STAT5 (94205S), GAPDH (8884S) from Cell Signalling Technology (CST, Beverly, MA, USA); rabbit monoclonal antibodies (mAbs) against JAK3 (ab45141), phospho-Tyr1022/1023 of JAK1 (ab138005), JAK1 (ab133666) from Abcam (Cambridge, UK).

The following antibodies were used in flow cytometry analysis: CD45 (#560178; BD Biosciences; #304037; BioLegend), CD56 (#562780, BD Biosciences; #392406, BioLegend; #IM2474, Beckman Coulter, Miami, FL, USA), CD3 (#300316; BioLegend), NKp46 (#331914; BioLegend), CD16 (#302012; BioLegend), CD34 (#343516; BioLegend), CD43 (#343206; BioLegend), IFN-*γ* (#502530; BioLegend), Ki67 (#350530; BioLegend), granzyme B (#561142; BD Biosciences), perforin (#353314; BioLegend), CD107a (#555801; BD Biosciences), 7-AAD antibodies (#559763; BD Bioscience), NKP30 (#130-112-430; Miltenyi, Bergisch Gladbach Germany), KIR2DL5A (#566330; BD Biosciences).

### 2.2 Cell lines

The NK92 NK cell line was cultured in RPMI 1640 medium enriched with 10% FBS, 400 U/mL IL-2, 1% MEM Non-Essential Amino Acids Solution, along with 1% penicillin, and 1% streptomycin. HEK293T, K562 and human hepatoma cell lines HCC-LM9 were grown in DMEM supplemented with 10% FBS, penicillin and streptomycin.

Purified human primary NK (PB-NK) cells were extracted from peripheral blood following previously established methods^17^. Blood samples from healthy individuals (age between 18 and 55 years) were collected from the Changsha Blood Center in China, under the approved protocol HCHLL-2023-182 by the Ethics Committee of Hunan Children's Hospital. Peripheral blood mononuclear cells were isolated from the blood leukocytes using a standard density gradient centrifugation method with Ficoll/Isopaque. The NK cells were then purified through negative selection with a commercial human NK cell isolation kit, after which they were subjected to fluorescence-activated cell sorting using the Aria III cell sorter from BD Biosciences. The purity of the sorted CD3^–^CD56^+^ NK cells exceeded 99.0%.

iPSC cell line, hiPSC-B1, was obtained from Cellapy and iPSC-NK cells were acquired after routinely differentiated from hiPSC-B1 as described previously^18^. Briefly, when the cells reached about 70% confluence, they were detached using TrypLE™ Select Enzyme and the reaction was halted with PGM1 medium. Subsequently, the cells were enumerated and resuspended at a density of 80,000 cells per well in a round-bottom 96-well plate, with each well containing 100 μL of STEMdiff™ APEL™2 medium, which was supplemented with 40 ng/mL SCF, 50 ng/mL VEGF 165, 20 ng/mL BMP4, 20 ng/mL FGF-basic, 10 ng/mL Activin A, and 10 μmol/L Y-27632. The peripheral wells of the plate were filled with sterile water to avert evaporation of the medium. The culture medium was replenished on a thrice-weekly basis. On Day 4 of EB differentiation, the culture medium was replaced with STEMdiff™ APEL™2 medium containing 50 ng/mL SCF, 20 ng/mL BMP4, 20 ng/mL FGF-basic, 100 ng/mL VEGF 165, 100 ng/mL IGF-II, and 3 μmol/L SB 431542. On the eighth day of the spin embryoid body (EB) differentiation process, cells from 14 to 16 wells of a 96-well plate were collectively transferred into one well of a 6-well plate that had been precoated with 2% gelatin and filled with 2 mL of NK differentiation medium, which supplemented with 50 ng/mL SCF, 20 ng/mL FGF-basic, 200 ng/mL VEGF 165, 20 ng/mL IGF-II, 25 ng/mL Flt3-Ligand, 25 ng/mL TPO, 50 ng/mL IL-3, 3 U/mL EPO, and 25 ng/mL IL-6. The NK cell culture medium was then refreshed every 3–4 days. The maturation of NK cells was assessed after a 28-day culture period.

The above cells were cultivated in a humidified, 5% CO_2_ incubator maintained at a constant temperature of 37 °C.

### 2.3 Cell counting assay

For loss-of-function assays, NK92-shCtrl/shmiR155HG (5 × 10^4^) cells were cultured in a 48-well plate for 72 h before assessment. Conversely, for gain-of-function experiments, iPSC cells were infected with lentivirus expressing the full-length miR155HG or its control sequence, then routinely differentiated into NK cells for 28 days before analysis.

### 2.4 Flow cytometry analysis

Cell staining was carried out as described previously^19^, ^20^, ^21^. For surface staining of CD45, CD56, CD3, NKP46, NKP30, KIR2DL5A and CD16, NK92 or iPSC-NK cells were incubated with the specified antibodies in 1 × phosphate-buffered saline (PBS) at room temperature (RT) for 15 min in a light-protected environment. For the detection of intracellular IFN-*γ*, iPSC-NK cells were treated with PMA in conjunction with BD Golgi Plug™ protein transport inhibitor for 6 h. Subsequently, the cells underwent staining using the Fixation/Permeabilization Solution Kit according to the manufacturer's protocol. For intracellular staining of Ki67, granzyme B and perforin, NK92 or iPSC-NK cells were treated with the Foxp3/Transcription Factor Staining Buffer Set to permeabilize them at 4 °C for 2 h. Subsequently, these cells were incubated with antibodies specific to Ki67, granzyme B, and perforin at RT for 30 min. For apoptosis staining of K562 cells, NK92-shCtrl/shmiR155HG cells, as well as iPSC-NK-Ctrl/miR155HG cells were harvested and stained with AnnexinV and 7-AAD antibodies suspended in 1 × binding buffer at RT for 15 min in the dark. The data were acquired on an LSRFortessa Flow Cytometer (BD Biosciences) and analyzed using FlowJo 10.5.3 software (Tree Star, Ashland, OR).

### 2.5 ELISA of IFN-γ secretion in cell culture medium

NK92 cell culture supernatant was harvested, and the levels of IFN-*γ* present in the supernatant were then quantified using a commercial ELISA kit, following the protocol provided by the manufacturer.

### 2.6 CD107a degranulation assay

The CD107a degranulation assay was carried out as described in previous studies^22^^,^^23^. In essence, NK92-shCtrl/shmiR155HG cells, stimulated with or without cytokines, were incubated with K562 cells at a ratio of 10:1 ratio in the presence of the anti-CD107a-PE antibodies and the protein transport inhibitor Golgi Plug and Golgi Stop. Following a 5-h co-incubation period at 37 °C, the proportion of CD107a-positive NK92 cells was examined using flow cytometry.

### 2.7 NK cell cytotoxicity assay

For K562 cells apoptosis assay, NK92-shCtrl/shmiR155HG cells, stimulated with or without cytokines, were incubated with CTV-labelled K562 at a ratio of 10:1 for 5 h. The cells were harvested and the frequencies of Annexin V-negative/7-AAD-negative, Annexin V-positive/7-AAD-negative, and Annexin V-positive/7-AAD-positive K562 cells were evaluated using the apoptosis kit by flow cytometry.

### 2.8 Model of human liver cancer and NK cell adoptive transfer

The mouse studies were approval by the Animal Ethics Committee of Hunan Children's Hospital, with the ethical approval number being HCHDWLL-2023-07. All procedures involving animals were conducted in compliance with the Guidelines for the Care and Use of Laboratory Animals as published by the National Institutes of Health (Publication No. 80-23, revised in 1996), as well as the institutional ethical standards for animal research.

NOD/ShiLtJGpt-Prkdcem26Cd52Il2rgem26Cd22Il15em1Cin(hIL15)/Gpt (NCG-IL15) mice (6 weeks of age) were utilized for the establishment of subcutaneous hepatocellular carcinoma (HCC) xenografts to evaluate the tumoricidal efficacy of NK92-shCtrl/shmiR155HG cells. Human HCC-LM9 cells (4 × 10^6^) were suspended in 100 μL of serum-free DMEM and injected subcutaneously into the inguinal region of the mice. One week post-inoculation, mice were intravenously administered with 2 × 10^6^ NK92-shCtrl/shmiR155HG cells *via* the tail vein on three separate occasions. After a total of 24 days from the initial injection, the mice were euthanized, and the resulting tumors were excised and weighed. The volume of the tumors at various time points was measured using a caliper and was determined by Eq (1):

(1) Volume = Length × Width^2^/2

### 2.9 RNA-sequencing and bioinformatics analysis

RNA was extracted from NK92-shCtrl/shmiR155HG cells stimulated with IL-2 (1000 U/mL) + IL-12 (10 ng/mL) for 12 h using AG RNAex Pro Reagent (AG21101; Accurate Biotechnology, Hunan, China). The quantity and purity of the RNA were assessed with BioDrop μLite equipment (Biochrom Ltd., Cambridge, UK). Poly(A) mRNA was purified using magnetic beads coated with Oligo(dT), and then the mRNA was randomly fragmented in a fragmentation buffer. Synthesis of the first strand of cDNA was carried out using the fragmented mRNA as a template and random hexamers for priming. Following end-repair and adapter ligation, RNA sequencing libraries were generated. These libraries were subsequently sequenced on an Illumina NovaSeq 6000 platform with 150 bp paired-end reads.

The raw sequencing reads were aligned to the human reference genome (hg38) using HISAT2 with default settings. StringTie (run with the “-e” parameter) and prepDE.py3 were used to calculate and merge read count information. Genes with a Counts per Million value greater than 1 in at least two samples were included in the analysis. Differential expression analysis was conducted using edgeR's exact test, with a logarithmic fold change (LFC) threshold set at log_2_(1.2) and a significance cutoff at a *P* value of less than 0.01. Gene Ontology (GO) enrichment analysis of DEGs was performed by clusterProfiler. The data presented in the figures are accessible in the published article and the Supporting Information The raw sequence data reported in this paper have been deposited in the Genome Sequence Archive in the National Genomics Data Center, China National Center for Bioinformation/Beijing Institute of Genomics, Chinese Academy of Sciences (GSA: CRA020503) that are publicly accessible at https://ngdc.cncb.ac.cn/gsa^24^^,^^25^.

### 2.10 Isolation of NK cells from HCC samples

Following the receipt of proper informed consent, fresh HCC tissues and their adjacent non-neoplastic liver tissues were collected from patients undergoing tumor resection at Hunan Provincial People's Hospital. The study protocol was approved by the Institutional Review Board of the same hospital (the ethical approval number 2023-151). To isolate NK cells from these human samples, the tissues were first rinsed with PBS, minced into smaller fragments, and then treated with a mixture of collagenase type II (1 mg/mL), collagenase type IV (1 mg/mL), and DNase I (0.01 mg/mL) in DMEM with 2.5% FBS for 45 min at 37 °C, as described previously^26^. The lymphocytes were then separated using a 30%–40% Percoll gradient and rinsed twice with PBS. Subsequently, CD45^+^CD56^+^CD3^−^NK cells were purified using a fluorescence-activated cell sorting Aria cell sorter from BD Biosciences.

### 2.11 Isolation of cytoplasm and nuclear fraction

Utilizing the NE-PER Nuclear and Cytoplasmic Extraction Reagents and adhering to the manufacturer's protocol, the cytoplasmic and nuclear RNA fractions were extracted from NK92 cells (1 × 10^7^). The purity of non-nuclear and nuclear fractions was determined using specific RNA markers, namely GAPDH for the cytoplasmic fraction and MALAT1 for the nuclear fraction^27^, respectively.

### 2.12 Analysis of gene expression

Quantitative real-time polymerase chain reaction (RT-qPCR) and Western blot analysis were conducted to assess the expression levels of the genes of interest. The primers used for RT-qPCR are listed in Supporting Information Table S1. The clone numbers for all antibodies used in our study have been added to Supporting Information Table S2.

### 2.13 RAP assay

The miRNAs associated with miR155HG were identified using an RAP Kit, following the guidelines provided by the manufacturer. Biotin-labeled miR155HG probes and scramble control probes were synthesized by BersinBio, and the complete list of probe sequences utilized in the RAP assay can be found in Table S1. The process commenced with the cross-linking of NK92 cells (5 × 10^7^) using formaldehyde, followed by lysis and sonication. Subsequently, the cell lysates were incubated with the probes for 4 h at 37 °C to facilitate hybridization. After hybridization, streptavidin beads were added to the mixture and allowed to rotate at RT for an additional h to capture the probe–miRNA complexes. The RNAs that were successfully bound to the beads were then eluted, subjected to purification, and prepared for subsequent RT-qPCR analysis to determine the miRNAs bound to miR155HG.

### 2.14 Transfection with miRNA mimics and inhibitors

As previously detailed^28^, NK92 cells were transfected with 200 nmol/L Cy3-conjugated miR-155/miR-6756 mimics or the negative control (NC), Cy3-conjugated miR-6756 inhibitor (anti-miR-6756) and a control inhibitor (anti-NC) without the use of a transfection reagent. Following a 48-h transfection period, the cells were collected by centrifugation at 200×*g* for 5 min, resuspended in PBS, and the Cy3-positive NK92 cells were isolated by cell sorting. The miRNA mimics and inhibitors employed in this study were procured from GenePharma (Shanghai, China), and their respective sequences are provided in Table S1.

### 2.15 ChIP assay

ChIP assays were conducted in accordance with the manufacturer's protocol for a ChIP assay Kit. NK92 cells were stimulated with IL-2 (1000 U/mL) and IL-12 (10 ng/mL) for 2 h before cross-linking with a 0.5% formaldehyde solution for 15 min at RT. Subsequently, the cells were sonicated to reduce DNA to fragments ranging from 200 to 750 base pairs. The chromatin was immunoprecipitated using 2 μg of anti-STAT3 antibody or a matched isotype control IgG (2729S, CST) at 4 °C for 16 h with agitation. Protein A/G MagBeads (88802, ThermoFisher Scientific) were then used for the enrichment of DNA-protein complexes. After thorough washing, the immunocomplexes bound to the beads were extracted with 420 μL of elution buffer (0.1 mol/L NaHCO_3_, 1% SDS) and agitated at RT for 1 h. The supernatants were then treated with Tris–EDTA buffer to reverse the cross-links between DNA and proteins, followed by an overnight incubation at 65 °C. The resulting DNA fragments were purified and analyzed by qPCR using primers detailed in Table S1.

### 2.16 Luciferase reporter assay

Luciferase reporter assay was carried out as described previously^29^. Cells were cultivated in a 48-well plate, each well containing 200 μL of complete growth medium. The luciferase activity was measured 48 h after transfection using the dual-luciferase reporter assay kit from Promega. The pRL-TK vector from Promega, which encodes for *Renilla* luciferase, was utilized as an internal control to normalize variations in transfection efficiency and cell harvesting.

To determine the impact of STAT3 expression on the transcriptional activity of the miR155HG promoter, HEK293T cells were co-transfected with 300 ng of either pcDNA 3.1 or pcDNA 3.1-STAT3 (Public Protein/Plasmid Library, Nanjing, China), 50 ng of the p(−2.5/+0.12 k) (TsingKe Biotech, Beijing, China) promoter construct, and 25 ng of the pRL-TK plasmid.

### 2.17 Public sequencing database

The correlation between the expression levels of various genes was assessed using Spearman's rank correlation coefficient, as derived from data obtained in GEPIA 2 (http://gepia2.cancer-pku.cn/#index) and the Gene Expression Omnibus (GEO) database (https://www.ncbi.nlm.nih.gov/gds/?term=). The putative binding sites of miRNAs in miR155HG and *JAK3* mRNA 3′UTR were predicted by TargetScan (https://www.targetscan.org/vert_71/). Transcription factors that may regulate miR155HG expression were predicted by ChIP-seq from the chEA3 (https://maayanlab.cloud/chea3/) website.

### 2.18 Statistical analysis

All statistical analyses were performed utilizing GraphPad Prism version 8.0, a software package by GraphPad Software Inc., based in La Jolla, CA, USA. The results are presented as the mean ± standard error of the mean (SEM), derived from a minimum of three separate experimental trials. For comparing differences among several groups, either two-tailed unpaired or paired Student's *t*-tests, or two-way ANOVA, were employed as appropriate. A statistical significance threshold was set at *P* < 0.05. Additionally, Spearman's correlation coefficient was applied to evaluate the correlation between expression levels of various genes.

## 3 Results

### 3.1 The levels of miR155HG expression exhibit a positive correlation with the activation and effector functions of NK cells

**Figure 1:**
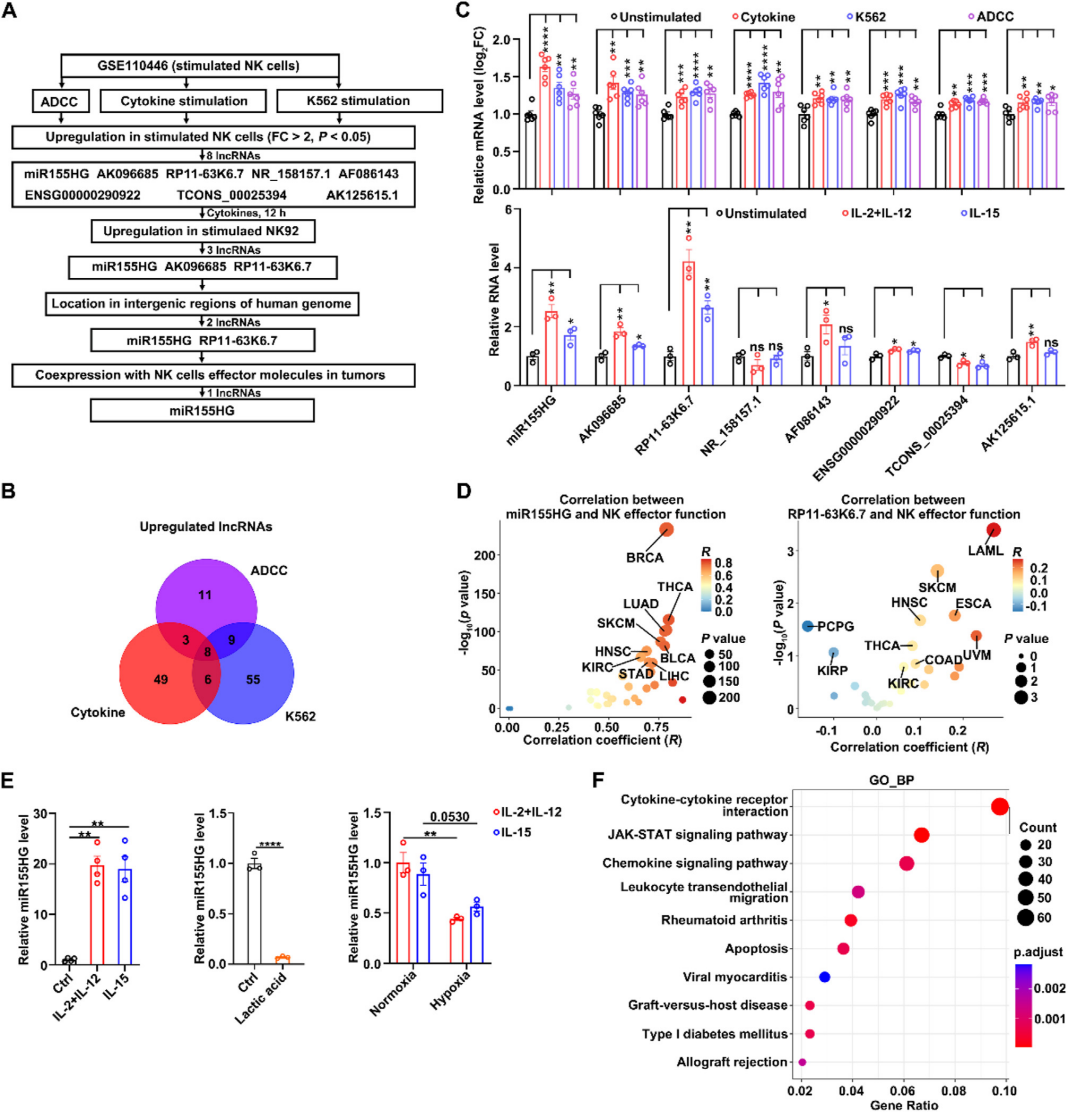
miR155HG levels are positively correlated with natural killer (NK) cell activation and effector functions. (A) The screening workflow for candidate long non-coding RNAs (lncRNAs) screening in activated NK cells using Gene Expression Omnibus (GEO) datasets (accession number: GSE110446). Cytokine: interleukin (IL)-12 and IL-18; ADCC: antibody-dependent cellular cytotoxicity; K562: an NK cell-sensitive tumor cell line. (B) Venn diagrams displaying upregulated lncRNAs of activated NK cells across three different stimulation conditions. (C) Top panel: the histogram shows the expression levels of the 8 upregulated lncRNAs in NK cells across three different stimulation conditions. Bottom panel: the expression levels of the 8 lncRNAs displayed in the top panel in NK92 cells stimulated with IL-15 alone or IL-2 plus IL-12 for 12 h. (D) Correlation between miR155HG and RP11-63K6.7 TPM (transcripts per kilobase of exon model per million mapped reads) and module TPM of NK effector function (*NCR1*, *EOMES*, *TBX21*, *CD69*, *DNAM1*, *KLRK1*, *PERF*, *IFNG*, and *GZMB*) in 33 different cancer types from the Cancer Genome Atlas Program (TCGA) datasets (http://gepia2.cancer-pku.cn/#index). Spearman's correlation coefficient (*R*) and *P* values are shown. (E) The expression levels of miR155HG in human primary NK (PB-NK) cells stimulated with IL-15 alone or IL-2 plus IL-12 for 12 h (left panel) or in NK92 cells treated with lactate (10 mmol/L) (middle panel) and hypoxia (1% O_2_) (right panel) for 12 h. (F) Gene Ontology (GO) analysis for the protein-coding genes co-expressed with miR155HG. The data from at least three independent experiments are presented as mean ± SEM (C, E); *P* values were assessed by unpaired Student's *t*-test (C, E). ∗*P* < 0.05; ∗∗*P* < 0.01; ∗∗∗*P* < 0.001; ∗∗∗∗*P* < 0.0001; ns, not significant. Abbreviations: ACC, adrenocortical carcinoma; BLCA, bladder carcinoma; BRCA, breast invasive carcinoma; CESC, cervical squamous cell carcinoma and endocervical adenocarcinoma; CHOL, cholangio carcinoma; COAD, colon adenocarcinoma; DLBC, lymphoid neoplasm diffuse large B-cell lymphoma; ESCA, esophageal carcinoma; GBM, glioblastoma multiforme; HNSC, head and neck squamous cell carcinoma; KICH, kidney chromophobe; KIRC, kidney renal clear cell carcinoma; KIRP, kidney renal papillary cell carcinoma; LAML, acute myeloid leukemia; LGG, brain lower grade glioma; LIHC, liver hepatocellular carcinoma; LUAD, lung adenocarcinoma; LUSC, lung squamous cell carcinoma; MESO, mesothelioma; OV, ovarian serous cystadenocarcinoma; PAAD, pancreatic adenocarcinoma; PCPG, pheochromocytoma and paraganglioma; PRAD, prostate adenocarcinoma; READ, rectum adenocarcinoma; SARC, Sarcoma; SKCM, skin cutaneous melanoma; STAD, stomach adenocarcinoma; TGCT, testicular germ cell tumors; THCA, thyroid carcinoma; THYM, thymoma; UCEC, uterine corpus endometrial carcinoma; UCS, uterine carcinosarcoma; UVM, uveal melanoma.

To identify lncRNAs elevated in activated NK cells, we conducted a bioinformatic analysis utilizing the 10.13039/100000085GEO dataset (GSE110446)^30^ and found that the lncRNA miR155HG fulfilled the following criteria (Fig. 1A–D; Supporting Information Fig. S1): (1) More than 2-fold upregulation in activated NK cells compared to unstimulated NK cells; (2) Found in intergenic areas of genome. (3) Coexpression with NK cells effector molecules in tumors^31^. These findings were also confirmed by both human PB-NK cells and NK92 cell lines. miR155HG expression was significantly increased in human PB-NK cells treated with IL-15 alone or a combination of IL-2 and IL-12 for 12 h, compared to unstimulated NK cells (Fig. 1E, left panel). Furthermore, lactate (Fig. 1E, middle panel) and hypoxia (Fig. 1E, right panel), which are two main suppressive factors in the tumor microenvironment, suppressed the expression of miR155HG in NK92 cells. Subsequent GO enrichment analysis of genes co-expressed with miR155HG indicated that miR155HG was highly co-expressed with cytokine–cytokine receptor interaction, JAK–STAT signaling pathway, and the chemokine signaling pathway (Fig. 1F). These findings indicate that miR155HG is elevated in activated NK cells and may be positively correlated with NK cell activation and the JAK–STAT signal pathway.

### 3.2 miR155HG promotes NK cell proliferation and survival

**Figure 2:**
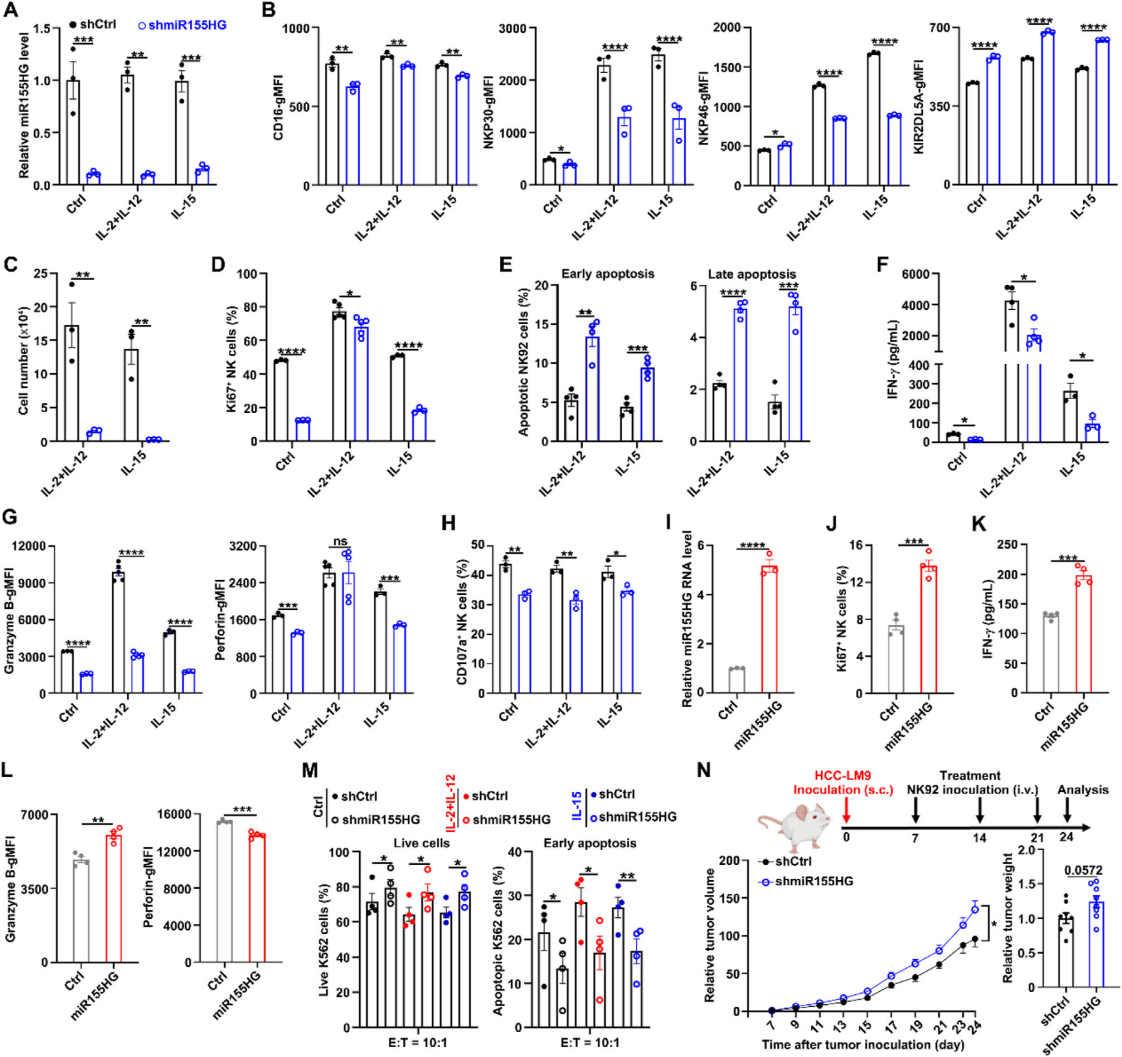
MiR155HG promotes NK cell proliferation, survival and effector functions. (A) Quantitative real-time polymerase chain reaction (RT-qPCR) assays of miR155HG levels in miR155HG knockdown (shmiR155HG) and control (Ctrl) NK92 cells with or without cytokine stimulation for 24 h. (B) Flowcytometry analysis of CD16, NKP30, NKP46 and KIR2DL5A expression on shmiR155HG and Ctrl NK92 cells after stimulated with or without cytokines for 24 h. gMFI: geometric mean fluorescence intensities. (C) Cell counting assay of shmiR155HG and Ctrl NK92 cells after stimulated with or without cytokines for 72 h. (D) Flowcytometry analysis of Ki67 expression on shmiR155HG and Ctrl NK92 cells after stimulated with or without cytokines for 24 h. (E) Flowcytometry analysis of the ratio of early apoptosis (Annexin V^+^7-AAD^–^) and late apoptosis (Annexin V^+^7-AAD^+^) of shmiR155HG and Ctrl NK92 cells after stimulated with cytokines for 48 h. (F) enzyme-linked immunosorbent assay (ELISA) of interferon-gamma (IFN-*γ*) in shmiR155HG and Ctrl NK92 cells after stimulated with or without cytokines for 24 h. (G) Flowcytometry analysis of the percentages of granzyme B and perforin in shmiR155HG and Ctrl NK92 cells after stimulated with or without cytokines for 24 h. (H) Flowcytometry analysis of the percentages of CD107a on shmiR155HG and Ctrl NK92 cells after co-cultured with K562 cells in the presence or absence of indicated cytokines for 5 h. (I) RT-qPCR assays of miR155HG levels in miR155HG overexpressed (miR155HG) and control (Ctrl) NK92 cells. (J) Flowcytometry analysis of Ki67 expression on miR155HG and Ctrl NK92 cells after stimulated with IL-2 (800 U/mL) for 24 h. (K) ELISA of IFN-*γ* in miR155HG and Ctrl NK92 cells after stimulated with IL-2 (800 U/mL) for 24 h. (L) Flowcytometry analysis of the percentages of granzyme B and perforin in miR155HG and Ctrl NK92 cells after stimulated with IL-2 (800 U/mL) for 24 h. (M) Flowcytometry analysis of the percentages of live (Annexin V^–^7-AAD^–^) and early apoptosis (Annexin V^+^7-AAD^–^) of K562 cells after co-cultured with shmiR155HG or Ctrl NK92 cells with or without cytokines for 5 h. (N) Mouse models of human HCC were established by subcutaneous injection of HCC-LM9 into NCG-IL15 mice. shmiR155HG and Ctrl NK92 cells were administered intravenously to recipients every 7 days starting at 7 days after HCC-LM9 inoculation (*n* = 8 mice/group). The relative tumor volume and the weight of excised tumors are shown. The relative values shown are fold change of tumor volume/weight at indicated times relative to the mean volume of shCtrl group on Day 7. The data from at least three independent experiments are presented as mean ± SEM (A–N); *P* values were assessed by unpaired (A–N) Student's *t*-test or two-way ANOVA (N). ∗*P* < 0.05; ∗∗*P* < 0.01; ∗∗∗*P* < 0.001; ∗∗∗∗*P* < 0.0001; ns, not significant.

We further evaluated whether miR155HG affected NK cell proliferation, activation and effector functions, using both the NK92 cell line and iPSC-NK cells. Firstly, we analyzed the expression levels of activating and inhibitory receptors on NK cell by flow cytometry. Compared with the control (shCtrl), the stable knockdown of miR155HG (shmiR155HG) (Fig. 2A) resulted in reduced geometric mean fluorescence intensities (gMFIs) of CD16, NKP30 and NKP46, whereas the gMFIs of KIR2DL5A were increased (Fig. 2B; Supporting Information Fig. S2A). Silencing of miR155HG (shmiR155HG) led to a decrease in the cell number (Fig. 2C) and the percentages of Ki67^+^ cells (Fig. 2D; Fig. S2B), but an increase in the ratio of early apoptosis (Annexin V^+^7-AAD^–^) and late apoptosis (Annexin V^+^7-AAD^+^) of NK92 cells (Fig. 2E; Fig. S2C). Subsequently, we determined INF-*γ* secretion using an ELISA assay on the supernatants from cultured NK92 stable cell lines after stimulated with or without cytokines for 24 h. We observed that the knockdown of miR155HG reduced INF-*γ* secretion, regardless of cytokine stimulation (Fig. 2F). Stable knockdown of miR155HG suppressed the gMFIs of granzyme B and perforin (Fig. 2G; Fig. S2D), irrespective of cytokine treatment. Knockdown of miR155HG also decreased the expression of CD107a, a biomarker indicative of NK cell degranulation in response to stimulation^32^, on NK92 cells when cocultured with K562 cells, with or without cytokines stimulation for 5 h (Fig. 2H; Fig. S2E). Consistently, the overexpression of miR155HG (miR155HG) (Fig. 2I) increased the percentages of Ki67^+^ cells (Fig. 2J; Fig. S2F), INF-*γ* secretion following stimulation with IL-2 for 24 h (Fig. 2K). Overexpression of miR155HG could increase the gMFIs of granzyme B without affecting the gMFIs of perforin after stimulation with IL-2 for 24 h (Fig. 2L; Fig. S2G). Furthermore, the suppression of miR155HG reduced the cytotoxic activity of NK92 cells against K562 cells. This was evidenced by an increased proportion of viable K562 cells (Annexin V^–^7-AAD^–^) and a decreased proportion of K562 cells undergoing early apoptosis (Annexin V^+^7-AAD^–^) after a 5-h co-culture with NK92 cells. These effects were observed irrespective of whether IL-15 or a combination of IL-2 and IL-12 was present (Fig. 2M; Fig. S2H).

*In vivo* studies involving subcutaneous injection of human hepatoma cells HCC-LM9 into humanized IL15 transgenic NCG (NCG-hIL15) mice demonstrated that weekly adoptive transfer (intravenously) of shmiR155HG-NK92 cells, beginning on Day 7 post-injection, resulted in faster tumor growth and greater tumor weight compared to mice that received shCtrl-NK92 cell (Fig. 2N). Collectively, our data demonstrate that miR155HG enhances the antitumor capability of NK cells.

### 3.3 miR155HG promoted hematopoiesis and iPSC-NK cell generation

**Figure 3:**
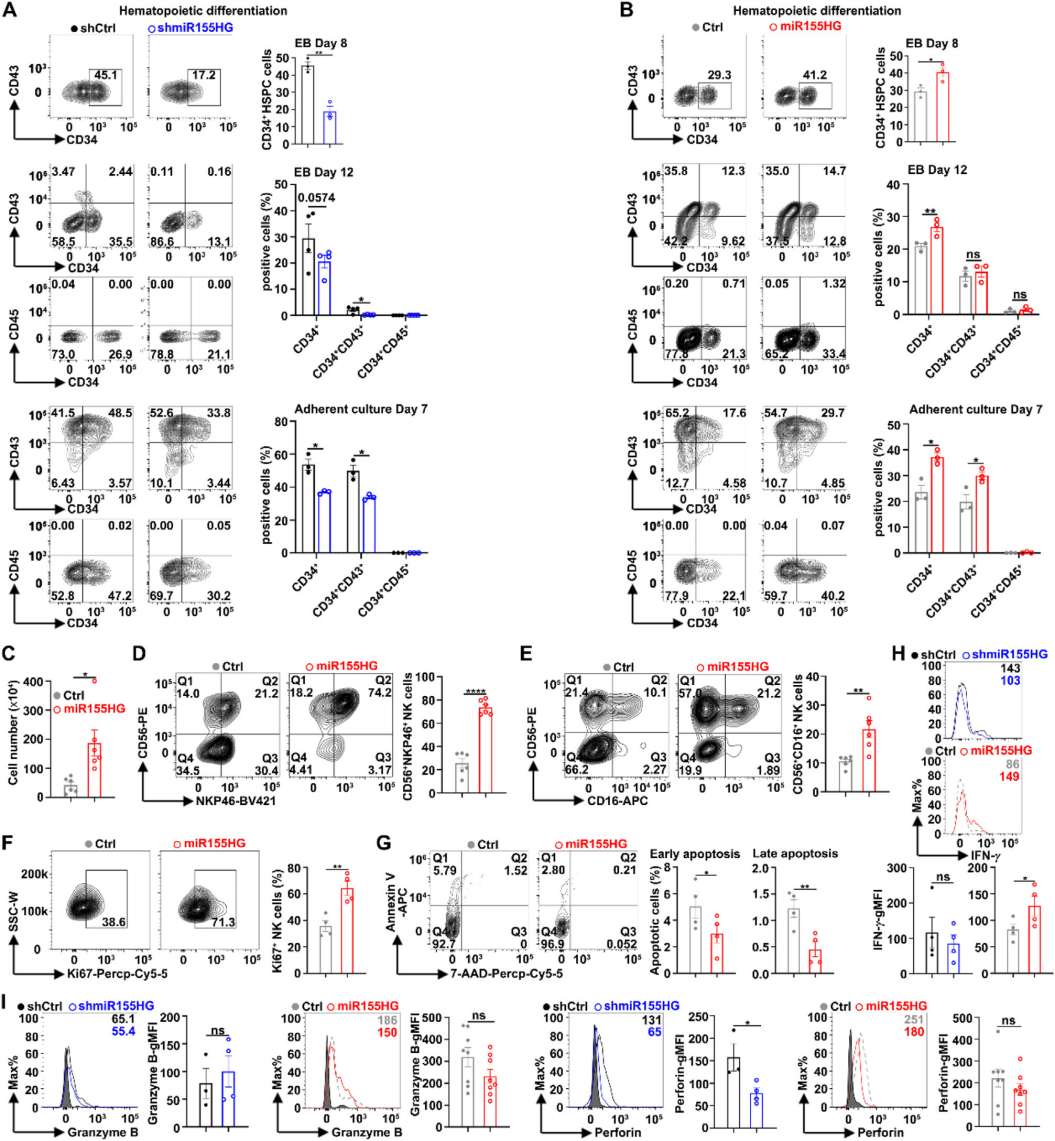
MiR155HG promoted hematopoiesis and iPSC-NK cell generation. (A, B) Flowcytometry analysis of the surface antigens of hematopoietic progenitor cells (CD34, CD43, and CD45) on Days 8 and 12 of embryoid body (EB) formation, and on Day 7 of adherent culture differentiation of iPSCs-shCtrl/shmiR155HG (A) or iPSCs-Ctrl/miR155HG (B). (C–G) Cell counting assay of total suspension cells (C), and flowcytometry analysis of the percentages of CD56^+^NKP46^+^iPSC-NK cells (D), CD56^+^CD16^+^iPSC-NK cells (E), Ki67^+^iPSC-NK cells (F) and early apoptosis (Annexin V^+^7-AAD^–^) and late apoptosis (Annexin V^+^7-AAD^+^) (G) in iPSC-NK cells at 28 days of iPSC-miR155HG and iPSC-Ctrl cells differentiation toward NK cells. (H, I) Flowcytometry analysis of the percentages of IFN-*γ* (H), granzyme B and perforin (I) in iPSCs-shCtrl/shmiR155HG or iPSCs-Ctrl/miR155HG after stimulated with IL-15 for 24 h. The data from at least three independent experiments are presented as mean ± SEM (A–I); *P* values were assessed by unpaired (A–I) Student's *t*-test. ∗*P* < 0.05; ∗∗*P* < 0.01; ∗∗∗∗*P* < 0.0001; ns, not significant.

Next, we examined the effects of miR155HG on NK cell differentiation, proliferation, activation, and effector functions in iPSC-derived NK cells. Human iPSCs were infected with lentivirus expressing full-length miR155HG or shmiR155HG sequences, as well as their control sequences, and routinely differentiated into hematopoietic progenitor cells and subsequently into NK cells, as previously reported^10^. To investigate the role of miR155HG during hematopoietic development, we analyzed the surface antigens of hematopoietic progenitor cells (CD34, CD43, and CD45) on Days 8 and 12 of EB formation, and on Day 7 of adherent culture differentiation of iPSCs into hematopoietic progenitor cells. Knockdown of miR155HG decreased the percentages of CD34^+^ cells and CD34^+^CD43^+^ HSPCs (Fig. 3A), while overexpression of miR155HG in iPSCs increased the percentages of these cells on Day 12 of EBs and this effect became more prominent on Day 7 of adherent culture (Fig. 3B). Overexpression of miR155HG in iPSCs increased the total number of iPSC-NK cells (CD45^+^CD56^+^) (Fig. 3C), as well as the percentages of CD56^+^NKP46^+^ iPSC-NK cells (Fig. 3D) and CD56^+^CD16^+^ iPSC-NK cells (Fig. 3E), and Ki67^+^ iPSC-NK cells (Fig. 3F), but decreased the proportions of both early and late apoptotic iPSC-NK cells (Fig. 3G) at 28 days of NK cell differentiation. These findings indicate that miR155HG can promote NK cell differentiation, proliferation, and survival.

We also examined the effect of miR155HG on the effector function of iPSC-NK cells. We found that the knockdown of miR155HG reduced and overexpression of miR155HG promoted the gMFIs of INF-*γ* (Fig. 3H). In the cases of miR155HG knockdown or overexpression, the gMFIs of Granzyme B remained unchanged (Fig. 3I, left panel). Knockdown of miR155HG can reduce the gMFIs of perforin, but overexpression of miR155HG does not increase the gMFIs of perforin (Fig. 3I, right panel).

### 3.4 miR155HG enhances JAK3 mRNA levels and the expression of downstream target genes of STAT

**Figure 4:**
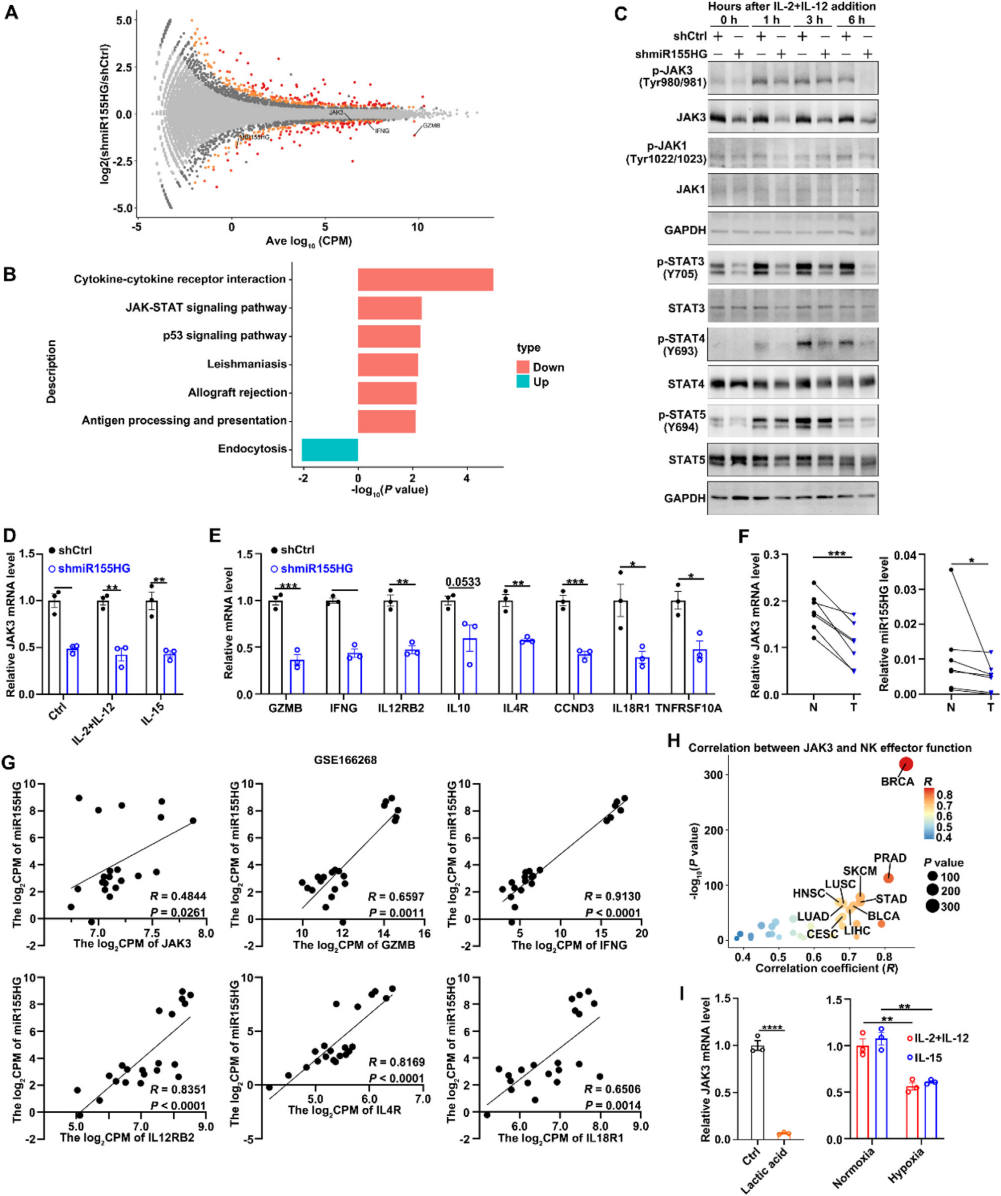
miR155HG promotes JAK3 expression and downstream STATs signaling. (A) The volcano plot displayed differentially expressed genes (DEGs) between shmiR155HG and Ctrl NK92 cells. (B) Up- and downregulated KEGG pathways of the DEGs between shmiR155HG and Ctrl NK92 cells. (C) Immunoblotting indicated the Janus kinase/signal transducers and activators of transcription (JAK–STAT) total and phosphorylated protein levels in shmiR155HG and Ctrl NK92 cells after simulated with IL-2 (1000 U/mL) + IL-12 (10 ng/mL) for the indicated times. (D, E) RT-qPCR analysis of *JAK3* (D) and JAK–STAT target genes (E) mRNA levels in shmiR155HG and Ctrl NK92 cells after stimulated with or without cytokines for 24 h. For (E), shmiR155HG and Ctrl NK92 cells were stimulated with IL-2 (1000 U/mL) + IL-12 (10 ng/mL). (F) RT-qPCR analysis of miR155HG and *JAK3* mRNA levels in NK cells isolated from HCC tumor tissues and noncancerous liver tissues. (G) Spearman's correlation coefficient analysis between miR155HG RNA level and the mRNA level of *JAK3* and its downstream target genes in NK cells from indicated GEO datasets. (H) Correlation between *JAK3* TPM and module TPM of NK effector function signature genes (*NCR1, EOMES*, *TBX21*, *CD69*, *DNAM1*, *KLRK1*, *PERF*, *IFNG*, and *GZMB*) in 33 different cancer types. The extensions of tumor abbreviations are listed in the legend of Fig. 1. (I) RT-qPCR analysis of *JAK3* mRNA levels in shmiR155HG and Ctrl NK92 cells after treated with lactate (10 mmol/L) and hypoxia (1% O_2_) for 12 h. For (D, E, I), the data from at least three independent experiments are presented as mean ± SEM. *P* values were assessed by unpaired (D, E, I) or paired (F) Student's *t*-test. ∗*P* < 0.05; ∗∗*P* < 0.01; ∗∗∗*P* < 0.001.

To further explore the mechanism underlying miR155HG promotes NK cell proliferation and effector function, we identified differentially expressed genes between shmiR155HG-NK92 and shCtrl-NK92 cell lines following treatment with IL-2 and IL-12 using bulk RNA-sequencing. After the suppression of miR155HG, 881 genes exhibited increased expression, while 583 genes showed decreased expression (Fig. 4A). The five most significantly downregulated KEGG pathways are cytokine–cytokine receptor interaction, the JAK–STAT signaling pathway, the p53 signaling pathway, leishmaniasis, and allograft rejection (Fig. 4B), and this finding aligns with the prior GO enrichment analysis of genes that are co-expressed with miR155HG (Fig. 1F). These results indicate that miR155HG may enhance NK cell activation and function by regulating the JAK–STAT signaling.

To test this hypothesis, we examined the levels of key components of the JAK–STAT pathway, including JAK1, JAK3, STAT3, STAT4, and STAT5, as well as their phosphorylation states after silencing miR155HG expression in NK92 cells. As shown, silencing miR155HG reduced the total protein level and phosphorylation level of JAK3, along with the phosphorylation levels of STAT3, STAT4, and STAT5, while the levels of JAK1 and phosphorylated JAK1 remained unchanged in NK92 cells after treatment with IL-2 plus IL-12 at different time points (Fig. 4C). The mRNA levels of *JAK3* and its downstream target genes, including *GZMB*, *IFNG*, *IL12RB2*, *CCND3*, *IL10*, *IL4R*, *IL18R1*, and T*NFRSF10A*, were downregulated in shmiR155HG NK92 cells, irrespective of cytokine stimulation (Fig. 4D and E).

To find more evidence supporting the positive regulation of miR155HG on *JAK3* mRNA levels, we also used several publicly available datasets as well as RT-qPCR of miR155HG and *JAK3* mRNA levels in tumor tissues from HCC patients. When compared with NK cells derived from healthy liver tissues, NK cells infiltrating tumors from patients with HCC showed reduced RNA levels for both *JAK3* and miR155HG (Fig. 4F). The expression of miR155HG exhibited a positive correlation with the mRNA levels of *JAK3* and its downstream target genes^33^ (Fig. 4G). The mRNA levels of *JAK3* also demonstrated a positive correlation with the functional module of NK cell effector functions across various human cancer types, as indicated by data from the TCGA and GTEx databases (Fig. 4H; Supporting Information Fig. S3). Moreover, lactate and hypoxia suppressed the mRNA expression of *JAK3* in NK92 cells (Fig. 4I).

These data indicate that miR155HG may promote NK cell activation and function by increasing *JAK3* mRNA levels and its downstream STAT signals.

### 3.5 miR155HG functions as a miR-6756 sponge to upregulate JAK3 mRNA levels

**Figure 5:**
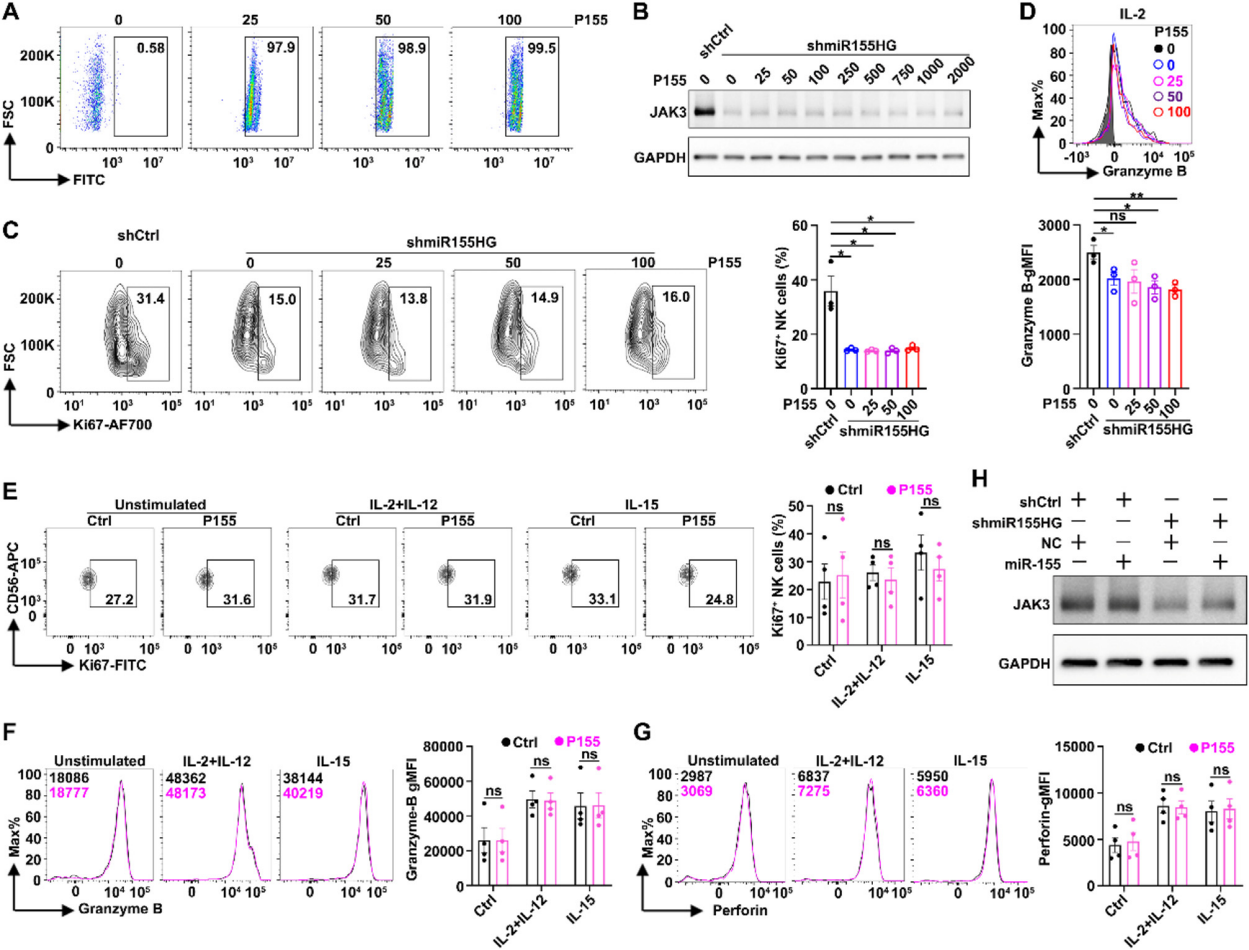
miR155HG may function as a lncRNA to upregulate JAK3 expression, irrespective of its derivative P155 and miR-155. (A–G) P155 failed to abrogate the inhibitory effect of miR155HG silencing on JAK3 expression as well as NK cell effector function-related molecules. (A) The efficiency of P155 entered NK92 cells. NK92 cells were treated with the indicated concentrations (μmol/L) of FITC-labelled P155 for 24 h before flowcytometry analysis. (B) NK92-shCtrl/shmiR155HG cells were treated with indicated concentrations of P155 for 48 h before immunoblotting or flow cytometry analysis. (C, D) Flowcytometry analysis of Ki67 (C) and granzyme B (D) expression on shmiR155HG and Ctrl NK92 cells treated with indicated concentrations (μmol/L) of P155 for 48 h. (E–G) The effect of P155 on the expansion and effector function of PB-NK cells. PB-NK cells stimulated with or without cytokines for 24 h and the expression of Ki67 (E), granzyme B (F) and perforin (G) was determined by flow cytometry. (H) miR-155 overexpression failed to reverse the shmiR155HG-induced suppression in JAK3 expression. NK92-shCtrl/shmiR155HG cells were transfected with 200 nmol/L Cy3 conjugated miR-155 mimic or its negative control (NC) for 48 h without transfection reagent and isolated Cy3^+^NK92 by fluorescence-activated cell sorting for immunoblotting. For (C–G), the data from at least three independent experiments are presented as mean ± SEM. *P* values were assessed by unpaired Student's *t*-test. ∗*P* < 0.05; ∗∗*P* < 0.01; ns, not significant.

We next explored how miR155HG enhances *JAK3* mRNA and protein levels. miR155HG is situated on chromosome 21q21 and comprises three exons encompassing a region of approximately 1.5 kilobases^16^. It is reported that miR155HG encodes a 17-amino acid micropeptide (P155)^16^ as well as a precursor RNA of miRNA-155^34^. Our data show that P155 neither abrogated the inhibitory effect of miR155HG silencing on JAK3 expression nor affected the expression of molecules related to NK cell proliferation and effector function (Fig. 5A–G). miR-155, a derivative of miR155HG, is reported to be upregulated during NK cell activation and induced IFN-*γ* production by inhibiting the expression of the inositol phosphatase SHIP1^35^. However, our data show that miRNA-155 overexpression did not rescue JAK3 protein levels in miR155HG knockdown NK92 cells (Fig. 5H).

**Figure 6:**
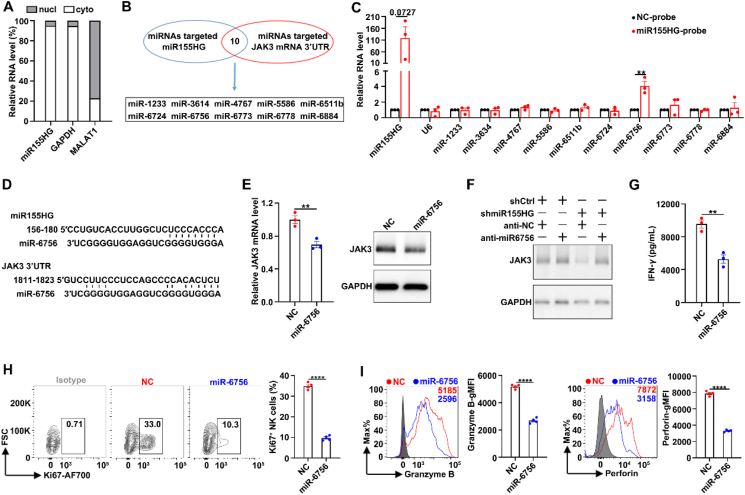
miR155HG functions as a miR-6756 sponge to upregulate *JAK3* mRNA levels. (A) The cytoplasm and nucleus distribution of miR155HG RNA in NK92 cells was determined by RT-qPCR analysis. Cyto, cytoplasm; nucl, nucleus. GAPDH and MALAT1 were used as positive controls for the fractions of cytoplasm and nucleus, respectively. (B) TargetScan prediction algorithm analysis of the potential binding miRNAs on both miR155HG and *JAK3* mRNA 3′UTR. (C) qPCR analysis of the 10 predicted miRNAs that bind to both miR155HG and *JAK3* mRNA 3′UTR in miR155HG precipitated RNAs. No error bar is shown for NC-probe groups because its normalized RNA level was set to 1. (D) The putative binding sites of miR-6756 in miR155HG and *JAK3* mRNA 3′UTR using TargetScan prediction algorithm analysis. Short vertical lines indicate the paired bases between miRNAs and their target sites in miR155HG and *JAK3* mRNA 3′UTR. (E) The mRNA and protein levels of *JAK3* in NK92 cells after transfected with miR-6756 mimic or its NC for 48 h. (F) The mRNA and protein levels of *JAK3* in shmiR155HG NK92 cells after transfected with miR-6756 inhibitor or its negative controls (anti-NC) for 48 h. (G–I) ELISA of IFN-*γ* (G) and flowcytometry analysis of the percentages of Ki67 (H), granzyme B (I, left panel) and perforin (I, right panel) in miR-6756 and NC NK92 cells after stimulated with IL-2 plus IL-12 for 24 h. For (C, E, G–I), the data from at least three independent experiments are presented as mean ± SEM; *P* values were assessed by unpaired (C, E, G–I) Student's *t*-test. ∗∗*P* < 0.01; ∗∗∗∗*P* < 0.0001; ns, not significant.

We next determined the cellular location of miR155HG and found it predominantly localized in the cytoplasm (Fig. 6A), suggesting that miR155HG may act as a miRNA sponge. Subsequent analysis with the TargetScan prediction algorithm identified 10 miRNAs with potential binding sites on both miR155HG and *JAK3* mRNA 3′UTR (Fig. 6B). To verify the direct interaction between miR155HG and miRNAs, biotinylated miR155HG and control probes were used to perform RAP assay with streptavidin beads. The data showed that, compared to the control probes, miR-6756, but not the other nine miRNAs, was significantly enriched in the miR155HG probes-precipitates (Fig. 6C). Analysis utilizing the TargetScan prediction algorithm revealed that miR-6756 potentially targets a single binding site within both the miR155HG and *JAK3* mRNA 3′UTR (1811–1823-nt) (Fig. 6D), and we then examined whether miR155HG upregulates JAK3 expression by working as a miR-6756 sponge. Overexpression of miR-6756 significantly suppressed both the mRNA and protein levels of *JAK3* in NK92 cells (Fig. 6E), whereas miR-6756 inhibitors rescued the reduced JAK3 protein levels in miR155HG-silenced NK92 cells (Fig. 6F). Moreover, we found that overexpression of miR-6756 could decrease the INF-*γ* secretion (Fig. 6G), the percentage of Ki67^+^ NK cells (Fig. 6H), and the gMFIs of granzyme B and perforin (Fig. 6I).

These results suggest that miR155HG enhances JAK3 protein expression by sequestering miR-6756, thereby reducing miR-6756's inhibitory effect on JAK3 expression and effector function of NK cells.

### 3.6 The transcription of miR155HG is induced by the cytokines–JAK–STAT3 axis

**Figure 7:**
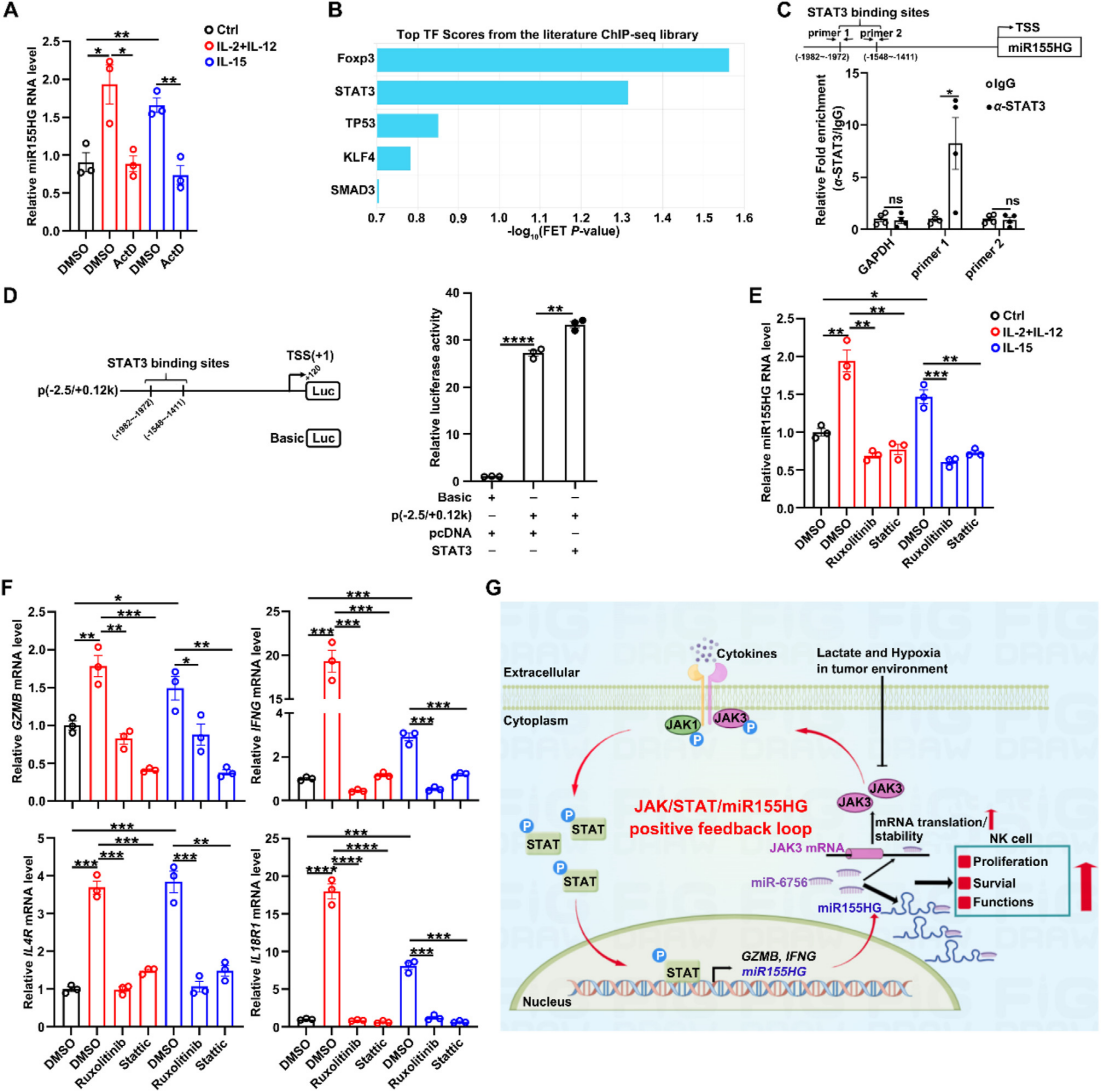
Identification of a JAK/STAT/miR155HG positive feedback loop in NK cell. (A) RT-qPCR assays of miR155HG levels after treatment of transcription inhibitor actinomycin D (ActD) with cytokines-stimulated NK cells. (B) Transcription factors that may regulate miR155HG expression were predicted using Chromatin immunoprecipitation (ChIP)-seq from chEA3 (https://maayanlab.cloud/chea3/). (C) Schematic structure of the miR155HG promoter with putative STAT3 binding sites (top panel) and ChIP assay of STAT3 binding to the miR155HG promoter in NK92 cells (bottom panel). The DNA precipitated by STAT3 antibodies was detected by PCR. The arrow designates the transcription direction of miR155HG. TSS, transcription start site. The promoter of GAPDH was used as a negative control. (D) The dual-luciferase reporter assay system was used to detect the effect of STAT3 overexpression on the activity of the miR155HG promoter vector. The arrow designates the transcription direction of miR155HG. TSS, transcription start site. (E, F) RT-qPCR of miR155HG and its downstream target genes RNA levels in NK92 cells after treated with the JAKs or STAT3 inhibitors with or without cytokines for 12 h. (G) The model of a JAK/STAT/miR155HG positive feedback loop and its role in NK cell expansion and effector functions (By Figdraw). For (A, C–F), the data from at least three independent experiments are presented as mean ± SEM; *P* values were assessed by unpaired Student's *t*-test (A, C–F). ∗*P* < 0.05; ∗∗*P* < 0.01; ∗∗∗*P* < 0.001; ∗∗∗∗*P* < 0.0001; ns, not significant.

We then explored the regulatory mechanism responsible for the upregulation of miR155HG in activated NK cells. It is widely recognized that cytokines, including IL-2, IL-12, IL-15, and IL-21, trigger NK cell activation through the JAK–STAT signaling pathway^6^. To examine whether cytokines promoted the expression of miR155HG at the transcriptional level, the expression of miR155HG after treatment of transcription inhibitor ActD with cytokines stimulated NK cells. We found that the role of cytokines in increasing miR155HG level was abrogated by blocking gene transcription with ActD (Fig. 7A), implying that cytokines may enhance miR155HG transcription. By using bioinformatics analysis based on ChIP-sequencing data, we identified potential binding sites for STAT3 within the core promoter region of miR155HG (Fig. 7B). Our ChIP assays revealed that the segment from −1981 to −1785 base pairs of the miR155HG promoter, but not the segment from −1735 to −1598 base pairs, was significantly enriched in the DNA precipitated by anti-STAT3 antibodies (Fig. 7C). To further validate the transcription factor STAT3 promoted miR155HG transcription, we constructed the promoter reporter p(−2.5/+0.12 k) of miR155HG (Fig. 7D, left panel) that exhibited much higher activity than the control plasmid pGL3-basic, suggesting that this segment contains the miR155HG promoter. Moreover, the promoter activity of p(−2.5/+0.12 k) was increased by overexpressing STAT3 (Fig. 7D, right panel). Furthermore, both the inhibitors of JAK and STAT3 significantly attenuated the upregulation of miR155HG (Fig. 7E) and its downstream target genes (Fig. 7F) induced by cytokines.

## 4 Discussion

The control of JAK–STAT signaling homeostasis is critical for maintaining both immune cells resting and immune response^36^^,^^37^. A previous study reported a negative feedback loop of JAK–STAT signaling in NK cells, where the IL-15*–*JAK–STAT signal axis induces upregulation of CIS, which interacts with JAK1, resulting in its enzymatic activity inhibition and proteasomal degradation^11^. In our research, we identified miR155HG as an activator of the JAK–STAT pathway in NK cells. JAK–STAT signaling triggered by cytokine stimulation led to the induction of miR155HG expression in NK cells. Mechanistically, upregulated miR155HG acts as a competing endogenous RNA partner of *JAK3* mRNA to maintain JAK3 levels in NK cells. Suppression of miR155HG led to decreased proliferation and survival of NK cells, as well as reduced IFN-*γ* secretion and cytotoxic capacity both *in vitro* and in vivo. Notably, overexpression of miR155HG promoted iPSC-NK cell expansion more than threefold, along with improved survival. These results strongly suggest that enhancing miR155HG expression provides a new target for NK cell adoptive transfer therapy, including improving the *in vitro* expansion efficiency and anti-tumor effect of NK cells.

Unlike other members of the JAK family, JAK3 is predominantly found in cells of hematopoietic origin and is recognized as the sole signaling component that directly engages with the *γ*c receptor^38^. Individuals with mutations in JAK3, whether human or murine, exhibit severe combined immunodeficiency, a condition defined by the absence of NK cells^7^^,^^39^^,^^40^. Prior research on mice carrying a spontaneous JAK3 mutation has unveiled a link between compromised JAK3 signaling and an arrest in the differentiation of NK cells and innate lymphoid cells 1 at the precursor NK and innate lymphoid cell precursor stages^41^. Downstream of the signal transduction of *γ*c cytokines, JAK3 phosphorylates STAT1/3/4/5/6, thereby regulating the downstream target genes. We found that miR155HG promotes the crucial target genes of JAK–STAT, including *GZMB*, *IFNG*, *IL12RB2*, *CCND3*, *IL10*, *IL4R*, *IL18R1* and *TNFRSF10A,* which contributed to NK cell expansion and effector function. Thus, our study identified miR155HG as a gatekeeper for the homeostatic expression of JAK3 in NK cells based on the following evidence: (1) Through bioinformatics analysis, RAP assay, and biochemical assays, it was discovered that miR155HG functions as an absorbent for miR-6756, thereby inhibiting the suppressive effects of miR-6756 on JAK3 expression. (2) In NK cells, the expression levels of miR155HG were found to be positively associated with the mRNA levels of *JAK3* and its target genes. In tumor-infiltrating NK cells derived from HCC patients, the RNA levels of both *JAK3* and miR155HG were observed to be downregulated. (3) According to data from the TCGA and GTEx project, the RNA levels of *JAK3* and miR155HG exhibited a positive correlation with the effector function module of NK cells across various human cancer types.

miR155HG exerts its biological function in a highly cell- and disease-dependent manner. In glioblastoma multiforme, miR155HG is overexpressed and works as a competing endogenous RNA for the tumor suppressor miR-185 to promote ANXA2 expression and tumor growth^42^. miR155HG exhibits elevated expression in dendritic cells during inflammation and encodes a 17-amino acid micropeptide, P155, that was previously unknown. This micropeptide P155 interacts directly with HSC70, thereby significantly enhancing autoinflammatory responses driven by dendritic cells in mice^16^. In this study, we found that the regulatory mechanism of miR155HG on JAK3 expression is independent of its derivative miR-155 and P155, as both failed to abrogate the inhibitory effect of silencing miR155HG on JAK3 expression. Furthermore, P155 did not influence the proliferation or the levels of effector molecules in NK cells. In this context, we discovered that miR155HG potentially functions as a sponge for miR-6756, inhibiting miR-6756's suppressive effect on JAK3 transcription, which in turn promotes the activation and effector functions of NK cells.

The blockade of SOCS proteins exerts a significant effect on strengthening NK cell-mediated tumor immunity, highlighting the potential of SOCS proteins as favorable targets for immunotherapeutic interventions. Lactate, a main suppressive factor in the TME, could suppress the expression of miR155HG and JAK3, attenuate the miR155HG/JAK/STAT positive feedback circuitry, and thereby reduce NK cell expansion and effector functions in the TME. Besides being a metabolite, lactate can induce gene expression through its receptor, which may be exploited for the induction of miR155HG expression in the TME by knocking in lactate response element in miR155HG promoter using gene editing. Lactate-induced-miR155HG-expressing NK cells may display more resistance to TME and enhanced tumor-killing ability.

## 5 Conclusions

In conclusion, we disclose that upon cytokine stimulation, STAT3 directly interacts with the miR155HG promoter and induces miR155HG transcription, and in turn, miR155HG functions by sequestering miR-6756, thereby preventing miR-6756 from repressing JAK3 expression. This sequence of events bolsters the JAK–STAT pathway, which is essential for NK cell development, activation, and function (Fig. 7G). Our findings underscore the crucial role of lncRNAs in modulating the cytokine/JAK/STAT signaling pathways and in driving the initiation and execution of NK cell responses. Furthermore, they indicate that these lncRNAs could serve as potential therapeutic targets for optimizing the outcomes of NK cell adoptive transfer treatments.

## Author contributions

Songyang Li: conceived and designed the experiments, curated and analyzed the data, acquired the funding, wrote original draft and revised the manuscript. Yongjie Liu, Peiwen Xiong: Data analysis. Xiaofeng Yin, Yao Yang, Jiaxing Qiu, Qinglan Yang, Xinjia Liu, Yana Li, Zhiguo Tan, Hongyan Peng, Shuting Wu, Xiangyu Wang and Lanlan Huang performed the experiments and analyzed the data. Sulai Liu, Yuxing Gong, Yuan Gao, Lingling Zhang, Junping Wang: analyzed the data, review and revised the manuscript. Yafei Deng and Zhaoyang Zhong: designed the research, supervised the study, and revised the manuscript. Youcai Deng devised the concept, designed the research, acquired the funding, supervised the study, wrote and revised the manuscript.

## Conflicts of interest

Songyang Li, Xiaofeng Yin, and Youcai Deng are beneficiaries of a pending patent for the recombinant plasmids and cells for iPSC-NK cell generation described in this manuscript.

## Appendix A Supplementary data

The following is the Supporting Information to this article.
