# Supplementary material for: Identification of a JAK–STAT–miR155HG positive feedback loop in regulating natural killer (NK) cells proliferation and effector functions
Source: Acta Pharm Sin B. 2025 Mar 2;15(4):1922–37. doi: 10.1016/j.apsb.2025.02.034 (PMC12138116; doi:10.1016/j.apsb.2025.02.034)
Supplement: Multimedia component 1 [file mmc1.pdf]

Supporting Information for

Original article

Identification of a JAK–STAT–miR155HG positive feedback loop in regulating natural killer (NK) cells proliferation and effector functions

Songyang Li[a,†], Yongjie Liu[a,†], Xiaofeng Yin[b,†], Yao Yang[b,c,†], Xinjia Liu[a,d], Jiaxing Qiu[e], Qinglan Yang[a], Yana Li[a], Zhiguo Tan[f], Hongyan Peng[a], Peiwen Xiong[a], Shuting Wu[a], Lanlan Huang[a,d], Xiangyu Wang[a], Sulai Liu[f], Yuxing Gong[g], Yuan Gao[g], Lingling Zhang[h], Junping Wang[i], Yafei Deng[a,*], Zhaoyang Zhong[j,*], Youcai Deng[b,i,*]

[a]*Pediatrics Research Institute of Hunan Province, the Affiliated Children's Hospital of Xiangya School of Medicine, Central South University (Hunan children's hospital), Changsha 410007, China*

[b]*Department of Clinical Hematology, College of Pharmacy and Laboratory Medicine Science, Army Medical University, Chongqing 400038, China*

[c]*Department of Pharmacy, the General Hospital of Western Theater Command of PLA, Chengdu 610083, China*

[d]*The School of Pediatrics, Hengyang Medical School, University of South China (Hunan Children's Hospital), Changsha 410007, China*

[e]*Department of Biological Sciences, Columbia University, NY 10027, USA*

[f]*Department of Hepatobiliary Surgery, Hunan Provincial People's Hospital (the First Affiliated Hospital of Hunan Normal University), Changsha 410005, China*

[g]*Translational Medicine Research Center, Shanxi Medical University, Taiyuan 030001, China*

[h]*Institute of Clinical Pharmacology, Anhui Medical University; Key Laboratory of Anti-inflammatory and Immune Medicine, Ministry of Education, Hefei 230032, China*

[i]*State Key Laboratory of Trauma and Chemical Poisoning, Institute of Combined Injury, Chongqing Engineering Research Center for Nanomedicine, College of Preventive Medicine, Army Medical University, Chongqing 400038, China*

[j]*The Fifth People's Hospital of Chongqing, Chongqing 400062, China*

Received 15 October 2024; received in revised form 1 January 2025; accepted 18 February 2025

*Corresponding authors.

E-mail addresses: yafeideng01@sina.com (Yafei Deng), zhongzhaoyang08@hotmail.com (Zhaoyang Zhong), youcai.deng@tmmu.edu.cn (Youcai Deng).

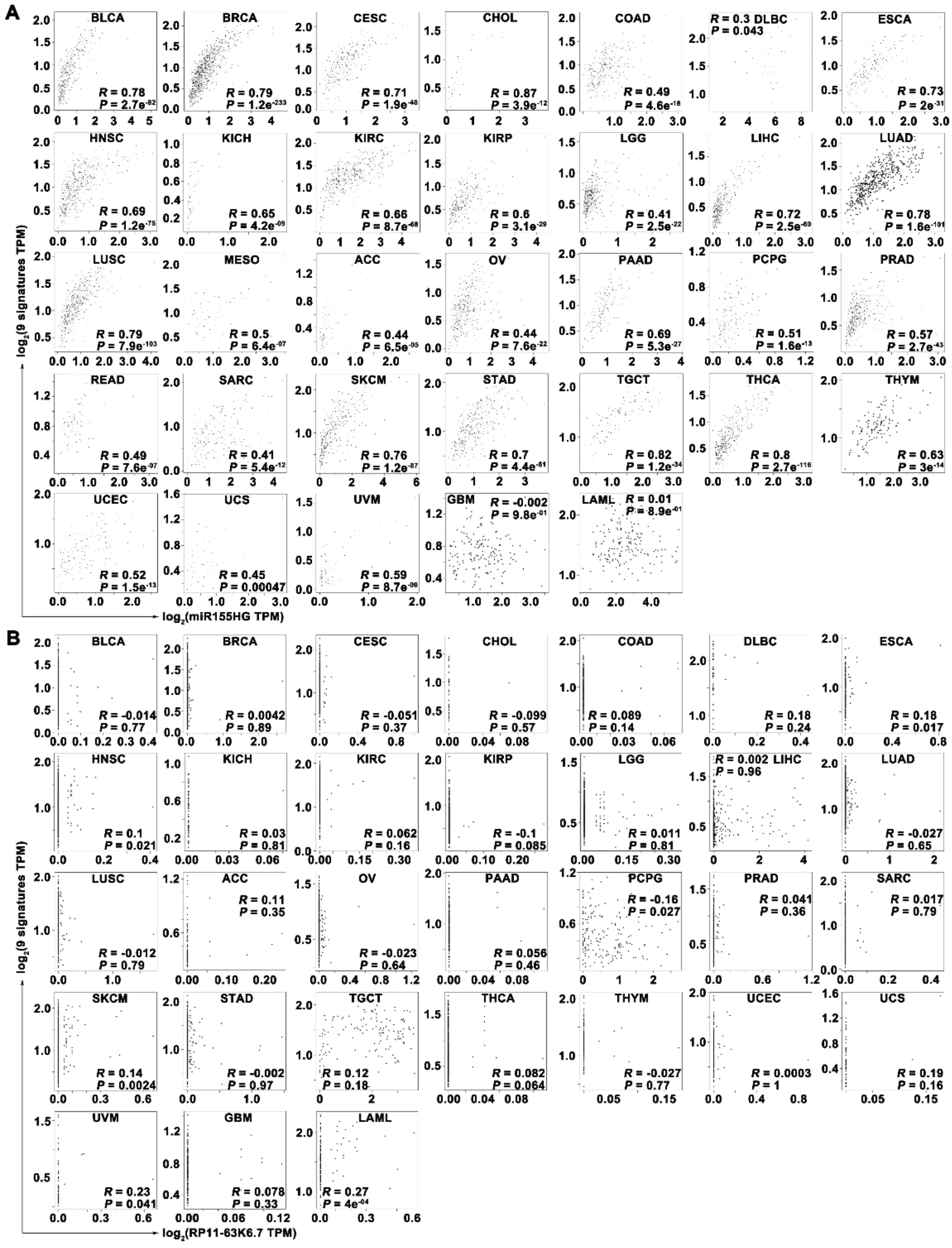

Figure S1 Correlation between miR155HG (A) or RP11-63K6.7 (B) TPM and module TPM of NK effector function related genes in 33 different cancer types. NK effector function related genes: *NCR1*, *EOMES*, *TBX21*, *CD69*, *DNAM1*, *KLRK1*, *PERF*, *IFNG*, and *GZMB*. Abbreviations: ACC, adrenocortical carcinoma; BLCA, adrenocortical carcinoma; BRCA, breast invasive carcinoma; CESC, cervical squamous cell carcinoma and endocervical adenocarcinoma; CHOL, cholangio carcinoma; COAD, colon adenocarcinoma; DLBC, lymphoid neoplasm diffuse large B-cell lymphoma,; ESCA, esophageal carcinoma; GBM, glioblastoma multiforme; HNSC, head and neck squamous cell carcinoma; KICH, kidney chromophobe; KIRC, kidney renal clear cell carcinoma; KIRP, kidney renal papillary cell carcinoma; LAML, acute myeloid leukemia; LGG, Brain lower grade glioma; LIHC,

liver hepatocellular carcinoma; LUAD, lung adenocarcinoma; LUSC, lung squamous cell carcinoma; MESO, mesothelioma; OV, ovarian serous cystadenocarcinoma; PAAD, pancreatic adenocarcinoma; PCPG, pheochromocytoma and paraganglioma; PRAD, prostate adenocarcinoma; READ, rectum adenocarcinoma; SARC, sarcoma; SKCM, skin cutaneous melanoma; STAD, stomach adenocarcinoma; TGCT, testicular germ cell tumors; THCA, thyroid carcinoma; THYM, thymoma; TPM, transcripts per kilobase of exon model per million mapped reads; UCEC, uterine corpus endometrial carcinoma; UCS, uterine carcinosarcoma; UVM, uveal melanoma. Spearman's correlation coefficient (*R*) and *P* values are shown.

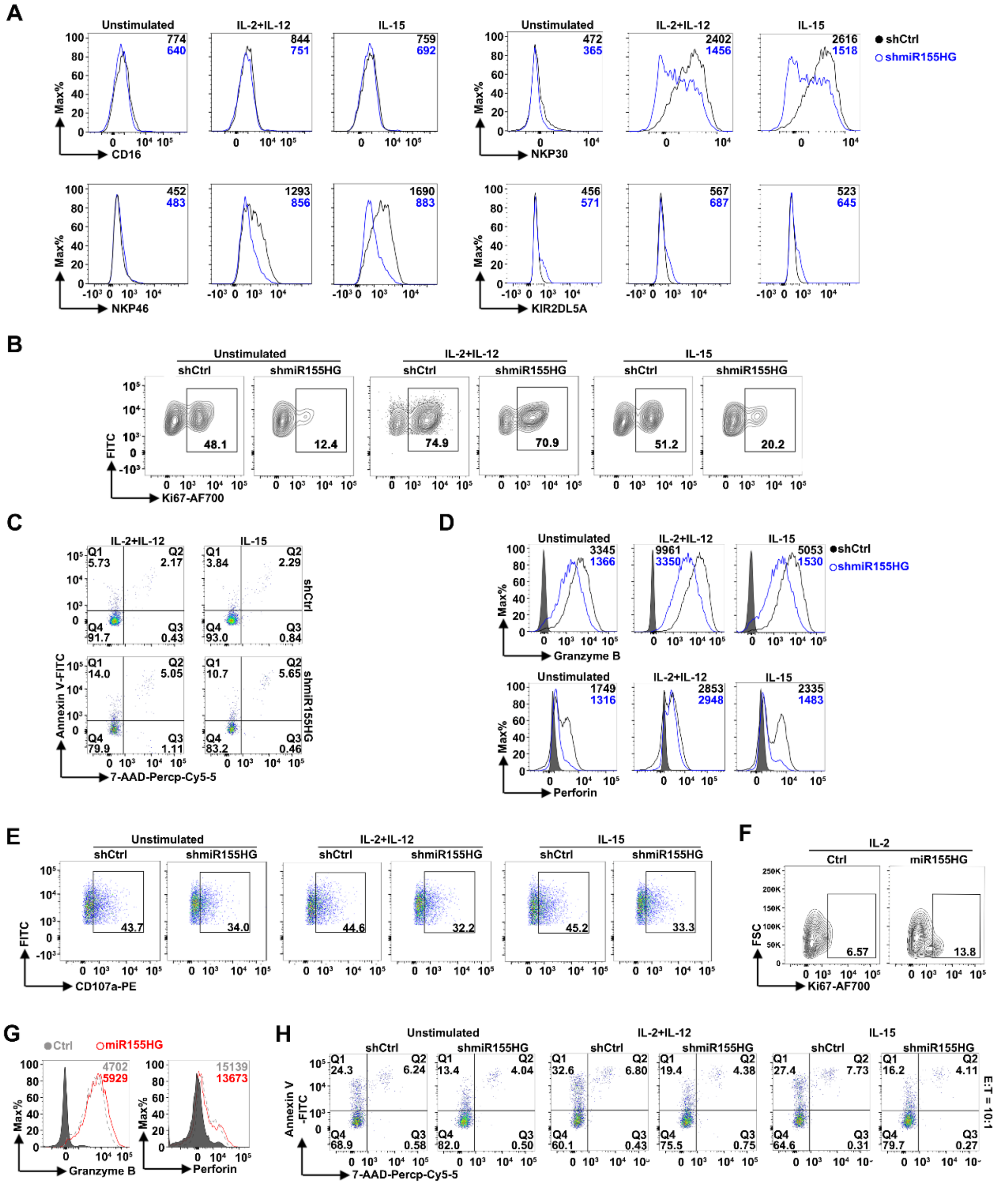

Figure S2 MiR155HG promotes NK cell growth and effector function. (A) Flowcytometry analysis of CD16, NKP30, NKP46 and KIR2DL5A expression on shmiR155HG and Ctrl NK92 cells after stimulated with or without cytokines for 24 h. gMFI: genomic mean fluorescence intensity. (B) Flowcytometry analysis of Ki67 expression on shmiR155HG and Ctrl NK92 cells after stimulated with or without cytokines for 24 h. (C) Flowcytometry analysis of the ratio of early apoptosis (Annexin V^{+}7-AAD^{-}) and late apoptosis (Annexin V^{+}7-AAD^{+}) of shmiR155HG and Ctrl NK92 cells after stimulated with cytokines for 48 h. (D) Flowcytometry analysis of the percentages of granzyme B and perforin in shmiR155HG and Ctrl NK92 cells after stimulated with or without cytokines for 24 h. (E) Flowcytometry analysis of the percentages of CD107a on shmiR155HG and Ctrl NK92 cells after co-cultured with K562 cells in the presence or absence of indicated cytokines for 5 h. (F) Flowcytometry analysis of Ki67 expression on miR155HG and Ctrl NK92 cells after stimulated with IL-2 (800 U/mL) for 24 h. (G) Flowcytometry analysis of the percentages of granzyme B and perforin in miR155HG and Ctrl NK92 cells after stimulated with IL-2 (800 U/mL) for 24 h. (H) Flowcytometry analysis of the percentages of live (Annexin V^{-}7-AAD^{-}) and early apoptosis (Annexin V^{+}7-AAD^{-}) of K562 cells after co-cultured with shmiR155HG or Ctrl NK92 cells with or without cytokines for 5 h.

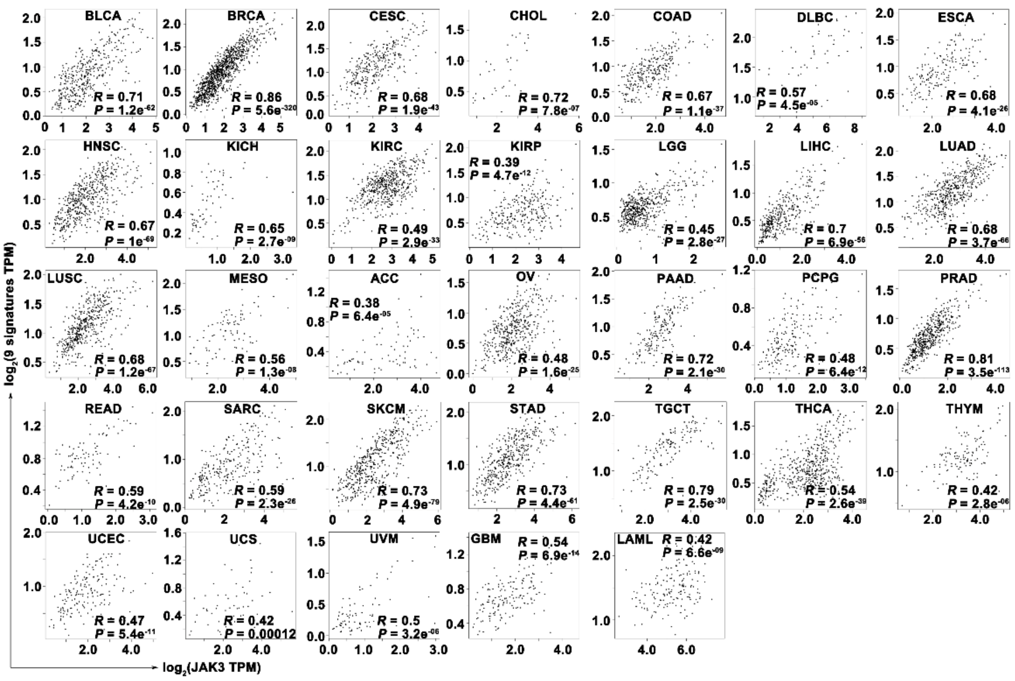

Figure S3 Correlation between JAK3 TPM and module TPM of NK effector function related genes in 33 different cancer types. The extensions of tumor abbreviations are listed in the legend of Fig. S1. Spearman's correlation coefficient (*R*) and *P* values are shown.

Table S1 Sequences of RNA and DNA oligonucleotides.

Name	Sense strand/Sense primer (5′–3′)	Antisense strand/Antisense primer (5′–3′)
Primers for qPCR		
miR155HG	TACCTGTCACCTTGGCTCTC	ACGGCAGCAATTTGTTCCAT
GAPDH	AGAAGGCTGGGGCTCATTTG	AGGGGCCATCCACAGTCTTC
JAK3	TTGGTTTGGGCTGGAGAAGT	TGACAGTCTTCAGCAGCTCT
AK096685	TGTCTCATCTGGTGCTCTTTCT	AACTGAACTGCCATAGAGCTT
RP11-63K6.7	CTCACGCATGTAATCCCTGC	TGGGTGTCTGTAGAGGGTGT
NR_158157.1	AACTGTACACCCACCCTCCT	AAATCCGGCTTGTCCCTCTG
AF086143	CTTGTGTGTCTGTGTGCGTG	GTTCACAGACACACACGCAC
ENSG00000290922	AGAGATGTGCCATGTCAGCC	ACAGTGAGCAGTTTCAGCCA
TCONS_00025394	TTGGAGAAAACGGTGGGGTT	CGGAAGCTGAAGTGGGAGAA
AK125615.1	AATGCTGCAGAGACCATGGG	ACTTCTGGCACACTGTTGGT
IFNG	TCGGTAACTGACTTGAATGTCCA	TCGCTTCCCTGTTTTAGCTGC
GZMB	CCCTGGGAAAACACTCACACA	GCACAACTCAATGGTACTGTCG
MALAT1	GAAAGCGAGTGGTTGGTAAA	CCCTCAAAAGCTTCAGACAA
IL12RB2	CCAGAGTTGATTGTTGATGGCA	CCTTGTCTGGGCTTCAAAGAG
IL10	CCGTGGAGCAGGTGAAGAAT	AGCCCCAGATCCGATTTTGG
IL4R	TGCGAGTGGAAGATGAATGGT	AGTTATCCGCACTGACCACG
CCND3	TGCACATGATTTCCTGGCCT	TTCAGTGCCAGTGATCCCTG
IL18R1	CTGATATCCCAGGCCACGTC	TGGGCAAAATCTCCACAGCA
IL18RAP	ATGCACAAAGTCCAGCGGTA	GCTCTGACTGTCCACGAACT
TNFRSF13C	GAGTGCTTCGACCTGCTG	CTCCAGCTCACCAGACCCA
XCR1	CCCAGAGAGCACCACCTTTT	CTGAGAGGCACAGGTTGAGG
CXCR6	TCAATGACAGCAGCCAGGAG	CCGTCAGGCTCTGCAACTTA
CCR1	CCAGAAGGTGAACGAGAGGG	CCAGGTTCAGGAGGTAGATGC
CCR6	TTTTCTGCCCACAATGAGCG	CTGGAGAACTGCCTGACCTC
TNFRSF10A	ACGAGATTCTGAGCAACGCA	CAGCAGCCTCCTCCTCTGA
U6	CTCGCTTCGGCAGCACA	AACGCTTCACGAATTTGCGA
hsa-miR-3614-3p		GCTAGCCTTCAGATCTTGGTGT
hsa-miR-4767		ATACGCGGGCGCTCCTGGCCGCCGCC
hsa-miR-5586-5p		GCCTATCCAGCTTGTTACTATATGC
hsa-miR-6511b-5p		AGGCAGAAGTGGGGCTGACA
hsa-miR-6724-5p		ATACTGGGCCCGCGGCGGGCGTGGGG
hsa-miR-6756-5p		TATAGGGTGGGGCTGGAGGT
hsa-miR-6773-5p		GGCCCAGGAGTAAACAGGAT
hsa-miR-6778-5p		TAGTGGGAGGACAGGAGGCA
hsa-miR-6884-5p		AGAGGCTGAGAAGGTGATGTTG
hsa-miR-1233-5p		ATTGGGAGGCCAGGGCA
**miRNA mimics**		
miR-155	UUAAUGCUAAUCGUGAUAGGGGUU	CCCCUAUCACGAUUAGCAUUAAUU
miR-6756	AGGGUGGGGCUGGAGGUGGGGCU	CCCCACCUCCAGCCCCACCCUUU
negative control (NC)	UGAAUUAGAUGGCGAUGUUdTdT	AACAUCGCCAUCUAAUUCAdTdT
**miRNA inhibitors**		
control inhibitor (anti-NC)	GUGGAUAUUGUUGCCAUCA	
anti-miR-6756	AGCCCCACCUCCAGCCCCACCCU	

**Primers for ChIP**		
p (−1982 to −1972)	GAGACATCATTATTGTCATT	TAGGAGTCAAATACACCTG
p (−1548 to −1411)	ATGGGAAATTCAGAAAGGC	TGATCATATGAGGGAGGAGC
GAPDH	TACTAGCGGTTTTACGGGCG	TCGAACAGGAGGAGCAGAGAGCGA
**Probes for RAP**		
miR155HG-1	CCTATCACGATTAGCATTAACAGCATACAGCCTACAG	
miR155HG-3	CAGACAATCCATGATATAACTGGAGGTTAGTAGTCCTTC	
miR155HG-4	ACCGTATTATGTGGCTAAGCTATTGAACTTGAACCTA	
miR155HG-5	ACTGCATAGAAATCCAAACCTTAAATATTCTGCAAATCAA	
Lac Z-1	GGGTTTTCCCAGTCACGACGTTGTAAAACGACGGCCA	
Lac Z-2	CGAAAGGGGGATGTGCTGCAAGGCGATTAAGTTGGGT	
Lac Z-3	GGAAGGGCGATCGGTGCGGGCCTCTTCGCTATTACGC	
Lac Z-4	TACCGCATCAGGCGCCATTCGCCATTCAGGCTGCGCA	
Lac Z-5	ACCATATGCGGTGTGAAATACCGCACAGATGCGTAAG	

Table S2 The information of antibodies used.

Antibody	Clone	Catalog number	Source
P-JAK3 (Tyr980/981)	D44E3	5031T	CST
JAK3	EP909Y	ab45141	Abcam
P-JAK1(Tyr1022/1023)	EPR1899	ab138005	Abcam
JAK1	EPR349	ab133666	Abcam
P-STAT3 (Tyr705)	D3A7	9145T	CST
STAT3	79D7	4904S	CST
P-STAT4 (Tyr693)	D2E4	4134S	CST
STAT4	C46B10	2653S	CST
P-STAT5 (Tyr694)	C11C5	9359S	CST
STAT5	D2O6Y	94205S	CST
GAPDH	D16H11	8884S	CST
CD45	2D1	560178	BD Biosciences
CD45	HI30	304037	BioLegend
CD56	NCAM16.2	562780	BD Biosciences
CD56	QA17A16	392406	BioLegend
CD56	N901	IM2474	Beckman Coulter
CD3	HIT3a	300316	BioLegend
CD16	3G8	302012/302018	BioLegend
NKP46	9E2	331914	BioLegend
CD34	581	343516	BioLegend
CD43	CD43-10G7	343206	BioLegend
Perforin	dG9	308122	BioLegend
Granzyme B	GB11	515406	BioLegend
IFN-γ	4S.B3	502530	BioLegend
Ki67	Ki-67	350530	BioLegend
Ki67	11F6	151222	BioLegend
Granzyme B	GB11	561142	BD Biosciences
Perforin	B-D48	353314	BioLegend

CD107a	H4A3	555801/328616	BD Biosciences
CD337 (NKP30)	REA823	130-112-430	Miltenyi
KIR2DL5A	UP-R1	566330	BD Biosciences
